# Menin-MLL1 complex cooperates with NF-Y to promote hepatocellular carcinoma survival

**DOI:** 10.1016/j.celrep.2025.116619

**Published:** 2025-11-25

**Authors:** Margarita Dzama-Karels, Mallory Sokolowski, Peyton Kuhlers, Jacqueline A. Brinkman, John P. Morris, Jesse R. Raab

**Affiliations:** 1 Department of Genetics, the University of North Carolina at Chapel Hill, Chapel Hill, NC 27599, USA; 2 Lineberger Comprehensive Cancer Center, the University of North Carolina at Chapel Hill, Chapel Hill, NC 27599, USA; 3 Department of Pharmacology, the University of North Carolina at Chapel Hill Medical School, Chapel Hill, NC 27599, USA; 4 Senior author; 5 Lead contact

## Abstract

Chromatin regulators are frequently mutated or aberrantly expressed in hepatocellular carcinoma (HCC), suggesting that the dysregulation of chromatin is a key feature driving liver cancer. In this study, using an epigenome-focused CRISPR screen in two-dimensional (2D) and three-dimensional (3D) conditions, we find the subunits of the menin-MLL1 complex to be among the strongest candidates for HCC survival. Inhibition of the menin-MLL1 interaction leads to global changes in occupancy of the complex with concomitant decreases in H3 lysine 4 trimethylation (H3K4me3), accessibility, and gene expression. Newly opened chromatin sites not bound by menin-MLL1 are associated with the recruitment of the pioneer transcription factor complex NF-Y yet remain embedded in silent chromatin domains, suggesting that they are primed for expression. A CRISPR-Cas9 screen of chromatin regulators in the presence of menin inhibitor SNDX-5613 reveals a significantly increased cell death when combined with *NFYB* knockout. Together, these data show that menin-MLL1 is necessary for HCC cell survival and cooperates with NF-Y to regulate oncogenic gene transcription.

## In brief

Dzama-Karels et al. identify the menin-MLL1 complex as crucial for hepatocellular carcinoma survival. Its disruption disturbs the PI3k/AKT/mTOR signaling pathway and chromatin accessibility. Menin inhibition promotes the redistribution of the NF-Y complex to sites associated with cell migration and invasion. Combining menin inhibition and *NFYB* knockout increases hepatocellular carcinoma cell death.

## Graphical Abstract

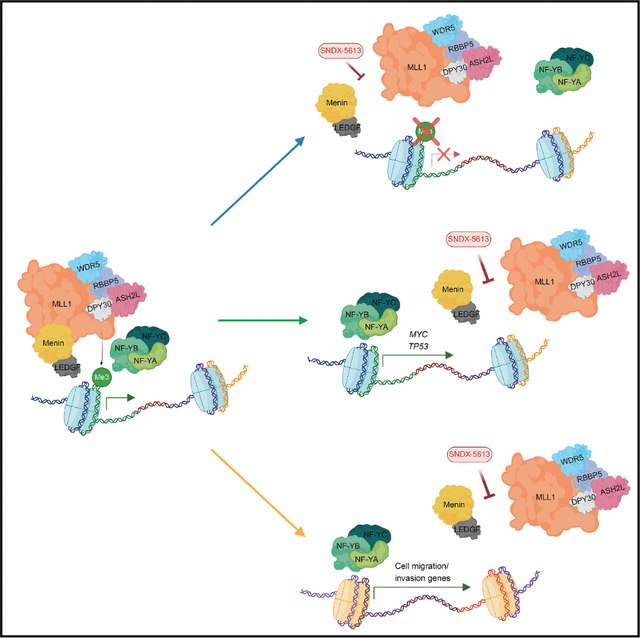

## INTRODUCTION

Liver cancer is the third leading cancer-related cause of death worldwide and the most rapidly growing cause of cancer deaths in the United States, with hepatocellular carcinoma (HCC) being the most frequent type of liver cancer (~80%).^1–3^ The 5-year relative survival rate of patients with liver cancer combining localized, regional, and distant stages is about 22%.^4,5^ Most patients are diagnosed at advanced stages of HCC, leaving them with systemic therapies and palliative options. Current first-line treatments include kinase inhibitors and immunotherapies that show only a modest survival increase.^2,6–9^ Identifying new vulnerabilities in HCC continues to be a critical medical need.

Aberrant genetic and epigenetic events drive the development and progression of HCC. Changes to the epigenome are proposed to induce cell plasticity and alter the fitness of cancer cells.^10^ Various epigenetic regulators, including lysine methyltransferases (EZH2, SETDB1, and EHMT2), demethylases (KDM3A, KDM4B, KDM5B, and KDM1A), and deacetylases (SIRT7 and HDAC1–3) were shown to be frequently upregulated in HCC.^11–13^ Notably, mutations in epigenetic modifiers occur in about 50% of HCC cases, highlighting the need to identify chromatin regulators essential for HCC survival in order to develop new targeted therapies.^14–20^

In this study, we used 2D and 3D CRISPR-Cas9 screens of genes involved in chromatin regulation to identify new vulnerabilities in HCC. Several of these targets are core subunits of the menin-MLL1 complex (*MEN1*, *ASH2L*, and *KMT2A*). Menin is a current therapeutic target in *MLL*-rearranged (*MLL*-r) and *NPM1-*mutant acute myeloid leukemias (AMLs), with one of the inhibitors, revumenib (SNDX-5613), approved by the United States Food and Drug Administration (FDA) for the treatment of relapsed or refractory *MLL*-r AML.^21^ The MLL1 protein is a “writer” of histone H3 lysine 4 trimethylation (H3K4me3), a mark associated with active promoters, and has also been suggested to be important in the proliferation of HCC and other cancers.^22–24^ Using a menin inhibitor and CRISPR-Cas9 knockout (KO) experiments, we systematically analyzed the global role of the menin-MLL1 complex on HCC survival by performing RNA sequencing (RNA-seq), cleavage under targets & release using nuclease (CUT&RUN), and assay for transposase-accessible chromatin using sequencing (ATAC-seq). These data revealed the HCC-specific oncogenic network regulated by the menin-MLL1 complex. We found that active promoters are bound by menin-MLL1 and the transcription factor (TF) complex nuclear transcription factor Y (NF-Y). Following menin inhibition, the menin-MLL1 complexes were depleted from chromatin, while NF-Y was lost from some menin-MLL1 targets and relocalized to new sites. A CRISPR-Cas9 screen of HCC cells in the presence of the menin inhibitor SNDX-5613 revealed potential partners for combined treatments, with the NF-YB TF among the top combinatorial hits. We show that the combined inhibition of the menin-MLL1 interaction and *NFYB* gene KO shows a significantly increased antiproliferative effect in HCC cells. Overall, this study defines the role of the menin-MLL1 complex in coordinating gene regulation to support the proliferation of HCC cells and defines new combinatorial approaches for future HCC therapy improvement.

## RESULTS

### Subunits of the menin-MLL1 complex are a vulnerability in HCC

**Figure 1.**
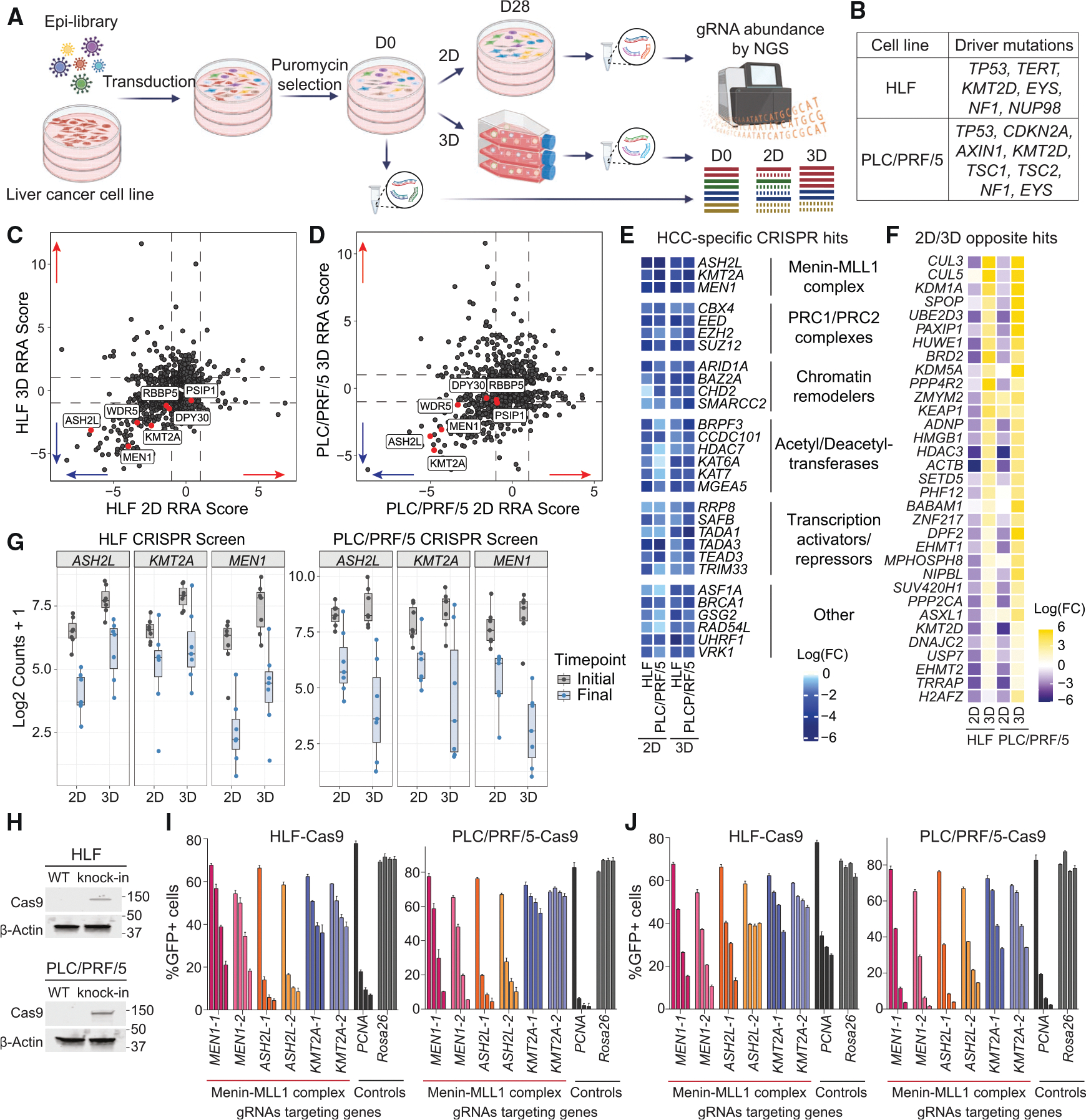
CRISPR-Cas9 screens identify the menin-MLL1 complex as HCC vulnerability (A) Scheme of epigenome-focused CRISPR-Cas9 screening in 2D/3D settings. (B) Summary of the most common mutations in HCC cell lines used for CRISPR-Cas9 screening. (C and D) Comparison of 2D/3D CRISPR screens in HLF (C) and PLC/PRF/5 (D) HCC cells using robust rank aggregation (RRA) scores. Subunits of menin-MLL1 are shown in red. (E) Heatmap of HCC-specific common negative hits among epigenetic regulators detected by CRISPR-Cas9 screens in HCC cell lines in 2D/3D settings. (F) Heatmap of hits showing opposite enrichment in 2D and 3D CRISPR-Cas9 screens in HCC cell lines. (G) Logarithmic counts of sequenced sgRNAs targeting *ASH2L*, *MEN1*, or *KMT2A* genes in 2D/3D screens in HLF (left) and PLC/PRF/5 (right) cells, comparing initial and final time points. (H) Immunoblotting against Cas9 and β-Actin proteins in HLF and PLC/PRF/5 parental and knockin cells. (I and J) Summary of negative-selection CRISPR-Cas9 screen with sgRNAs targeting the *MEN1*, *ASH2L*, or *KMT2A* gene and *PCNA* or *Rosa26* serving as the positive or negative controls, respectively, in 2D (I) and 3D (J) depicted at days 3, 14, 21, and 28. Bar graphs represent the mean of measurements of 3 independently transduced sets of cells. Error bars represent the standard deviation (SD).

To identify new potential targets of HCC among epigenetic regulators, we constructed a CRISPR library of ~6,000 guide RNAs (gRNAs) targeting 737 genes involved in chromatin-mediated gene regulation with 7 gRNAs targeting each gene and containing 855 non-targeting controls (NTCs; Table S1). Using this library, we performed an epigenome-focused CRISPR-Cas9 screen on two HCC human cell lines (HLF and PLC/PRF/5) in two-dimensional (2D) monolayer and three-dimensional (3D) spheroid settings for 28 days (Figures 1A and 1B; Table S2). For 3D conditions, cells were grown on low-adherent plates in methylcellulose-containing media (0.75%) to prevent attachment. These conditions have been shown to better mimic *in vivo* growth and revealed distinct vulnerabilities between 2D and 3D CRISPR-Cas9 screens.^25^ The changes in gRNA representation were calculated relative to the initial time point (day 0), reflecting the targets that affect the survival and proliferation of HCC cells (Figures 1C and 1D). A comparison of the 2D and 3D scores revealed a select group of genes essential in both conditions (Figures 1C, 1D, S1A, and S1B). The gRNAs showing the highest negative scores, indicating the decrease in cell survival upon a gene KO, were then compared to the Cancer Dependency Map (DepMap24Q4 Public^26,27^) to eliminate commonly essential genes. The common negative hits in all of the performed CRISPR-Cas9 screens included members of the menin-MLL1 complex (*ASH2L*, *KMT2A*, and *MEN1*), PRC1/PRC2 complexes (*CBX4*, *EED*, *EZH2*, and *SUZ12*), chromatin remodelers (*ARID1A*, *BAZ2A*, *CHD2*, and *SMARCC2*), acetyl- and deacetyl-transferases (*BRPF3*, *CCDC101*, *HDAC7*, *KAT6A*, *KAT7*, and *MGEA5*), transcriptional activators or repressors (*RRP8*, *SAFB*, *TADA1*, *TADA3*, *TEAD3*, and *TRIM33*), and other hits (*ASF1A*, *BRCA1*, *GSG2*, *RAD54L*, *UHRF1*, and *VRK1*) and represent potential targets for primary HCC (Figure 1E). As several subunits from the menin-MLL1 complex were identified as negative regulators of HCC survival in both 2D and 3D CRISPR-Cas9 screens and the function of this complex as a unit has not been previously studied in HCC, we decided to focus on deciphering its role in regulating transcription and chromatin in HCC.

For some genes, we observed dramatic differences between 2D and 3D screens in HLF and PLC/PRF/5 cells, consistent with recent work in lung adenocarcinoma (Figures 1F, S1C, and S1D^25^). We detected 33 genes showing opposite enrichment in 2D and 3D screens in HLF cells, including *KEAP1*, a known tumor suppressor that also mediates sorafenib, lenvatinib, and regorafenib resistance.^28,29^ Multiple genes involved in ubiquitination (*CUL3*, *CUL5*, *UBE2D3*, *HUWE1*, and *SPOP*) showed a proliferation benefit or were neutral only when knocked out in 3D CRISPR screens, while they were highly essential in 2D conditions, suggesting that ubiquitination plays a restrictive role in HCC growth in 3D conditions. Notably, *CUL3*, *UBE2D3*, and *HUWE1* are all considered common essential genes in DepMap24Q4. These findings support the importance of CRISPR screening in the 3D spheroid model and highlight the impact of growth conditions on phenotype.

When analyzing the individual gRNA results for members of the menin-MLL1 complex, we confirmed that gRNAs targeting multiple complex members were depleted in both 2D and 3D conditions (Figure 1G). To validate the importance of the menin-MLL1 complex members in the orthologous assay, we performed a CRISPR-Cas9 competitive growth assay using two gRNAs each for *MEN1*, *ASH2L*, *KMT2A*, and *KMT2B* genes with *PCNA* as a positive control and *Rosa26* as a negative control in Cas9-expressing HLF and PLC/PRF/5 cells under both 2D and 3D growth conditions (Figures 1H–1J and S1E–S1I). These data showed a strong dependency of HCC cells on *MEN1* and *ASH2L* genes and less on *KMT2A* and *KMT2B*. Since both MLL1 and MLL2 proteins interact with menin and WDR5-RbBP5-ASH2L-DPY30 (WRAD) subunits, they may compensate for each other in the absence of one of them. These findings confirm the importance of menin and ASH2L as a part of the menin-MLL1 complex in HCC cell growth, suggesting that this complex is necessary for HCC survival.

### Menin inhibition strongly affects localization and activity of the menin-MLL1 complex on chromatin

**Figure 2.**
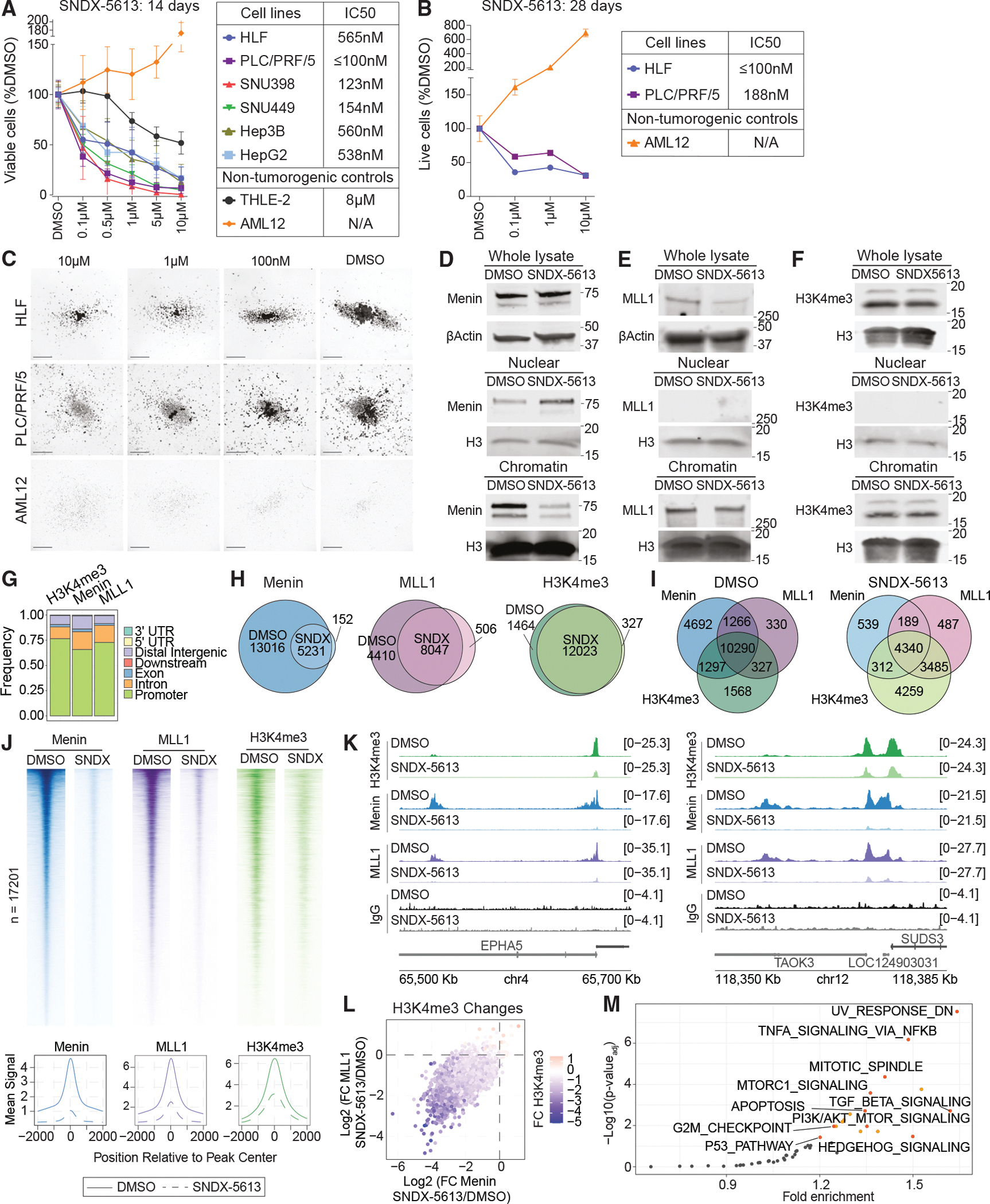
Inhibition of the menin-MLL1 interaction affects HCC cell survival by perturbing the complex binding to sets of genes (A) HCC and liver cell viability following SNDX-5613 treatment assessed by CellTiter-Glo (CTG) assay after 14 days in 2D. Error bars represent the SD of 3 independent experiments, each performed in 4 technical replicates. (B and C) HCC and murine liver cell viability and growth after 28 days of SNDX-5613 treatment in 3D media. Cell viability was measured using the CTG assay (B), and cell growth and colony formation ability were assessed by microscopy (C). Micrographs were taken at ×1.25 amplification, with the bar indicating 1.3 mm. (D–F) Immunoblotting against menin (D), MLL1 (E), and H3K4me3 (F) with corresponding controls, such as β-Actin or total H3, in HLF cells following treatment with 5 μM SNDX-5613 for 4 days. (G) Distribution of binding sites relative to genomic features for H3K4me3, menin, and MLL1. (H and I) Venn diagrams of detected independent (H) or overlapping (I) menin, MLL1, and H3K4me3 peaks in HLF cells following treatment with DMSO or 5 μM SNDX-5613 (SNDX) for 4 days. Occupancy peaks for all the groups are selected based on menin binding sites. (J) Heatmaps showing the correlation of promoter peaks in a ±2-kb window with occupancy of H3K4me3, menin, and MLL1 across CUT&RUN data from HLF cells treated with 5 μM SNDX-5613 for 4 days. The bottom metaplots show the mean of overall peak signals detected at the regions comparing DMSO and SNDX-5613 treatments in HLF cells. (K) Examples of CUT&RUN tracks showing distribution of H3K4me3, menin, MLL1, and immunoglobulin (Ig)G binding to the promoter regions in HLF cells treated with 5 μM SNDX-5613 for 4 days. (L) Logarithmic fold changes (FCs) of the H3K4me3 mark, menin, and MLL1 protein binding to the menin sites comparing conditions of HLF cells treated with 5 μM SNDX-5613 to those treated with DMSO for 4 days. (M) GREAT analysis of the menin-MLL1 complex targets using the hallmark gene set as a reference, comparing HLF cells treated with SNDX-5613 to those treated with DMSO.

To evaluate HCC cell survival by inhibiting the menin-MLL1 interaction without requiring a complete KO of the *MEN1* gene, we utilized the publicly available small molecule SNDX-5613 (revumenib). After treating cells for 14 days in 2D and 28 days in 3D, we observed a profound dose-dependent cell viability reduction in all tested HCC cells (HLF, PLC/PRF/5, SNU398, SNU449, HepG2, and Hep3B), whereas a normal human and a murine liver epithelial cell line, THLE-2 and AML12, showed only a slight change in growth with higher concentrations of SNDX-5613 treatment (Figures 2A–2C and S2A–S2D). Detailed analysis of Annexin V staining in HCC cells upon SNDX-5613 treatment has not revealed significant differences, but a small effect on cell cycle progression by propidium iodide staining suggested cell death through a non-programmed or cell arrest mechanism (Figures S2E–S2G). These results support the use of menin inhibitors to dissect the function of menin-MLL1 in HCC.

Next, we evaluated the impact of menin inhibition on menin-MLL1 binding to chromatin. We treated HLF cells with 5 μM SNDX-5613 for 4 days, when cells largely remained viable, allowing us to separate the chromatin and transcriptional changes induced by menin inhibition rather than cellular death (Figure S2B). While total menin protein levels remained unchanged, we observed its robust removal from chromatin and accumulation in the nuclear fraction as assessed by immunoblotting (Figure 2D). MLL1 protein was reduced upon SNDX-5613 treatment in total protein, while the global levels of the H3K4me3 mark did not show any visible decrease (Figures 2E, 2F, and S2H).

Next, we assessed the impact of menin inhibition on the genomic occupancy of menin and MLL1 proteins and H3K4me3 distribution using CUT&RUN.^30^ In HLF cells treated with vehicle, we identified 28,123 menin, 17,791 MLL1, and 13,739 H3K4me3 peaks, with the majority localized to promoter regions (Figures 2G and 2H; Table S3). Comparing peaks in the DMSO- and SNDX-5613-treated conditions showed a decrease in the menin and MLL1 peak numbers after SNDX-5613 treatment, while H3K4me3 peaks were largely unchanged (Figure 2H). Notably, the binding sites of menin, MLL1, and H3K4me3 showed significant overlap, consistent with the established role of menin-MLL1 in H3K4me3 methylation at promoters (Figure 2I). Following treatment with 5 μM SNDX-5613 for 4 days, we noted a ~90% reduction in the menin signal, a ~3-fold reduction in MLL1, and a ~2-fold reduction in H3K4me3 (Figures 2J–2K and S2I). Differential occupancy analysis confirmed the overall decrease in menin binding as well as a reduction in the magnitude of MLL1 binding and H3K4me3 (Figure S2J). Most sites exhibited a coordinated decrease in menin, MLL1, and H3K4me3 signals, while fewer sites maintained a stable level of H3K4me3 even after the menin-MLL1 complex was removed from the region (Figures 2L and S2K). To evaluate pathways regulated by menin-MLL1 in HCC, we performed ontology analysis using the Genomic Regions Annotation Tool (GREAT^31^) of gene promoters directly bound by both menin and MLL1 proteins and identified enrichment for the tumor necrosis factor alpha (TNF-α), transforming growth factor β (TGF-β), MTORC1, PI3K/AKT, and Hedgehog signaling pathways (Figure 2M; Table S4). Together, these data suggest that menin-MLL1 regulates a large portion of the transcriptionally active regions of the genome that are enriched for genes involved in signaling and cell survival pathways necessary for HCC cell growth.

### Disruption of menin deregulates expression of critical pathways for HCC signaling pathways

**Figure 3.**
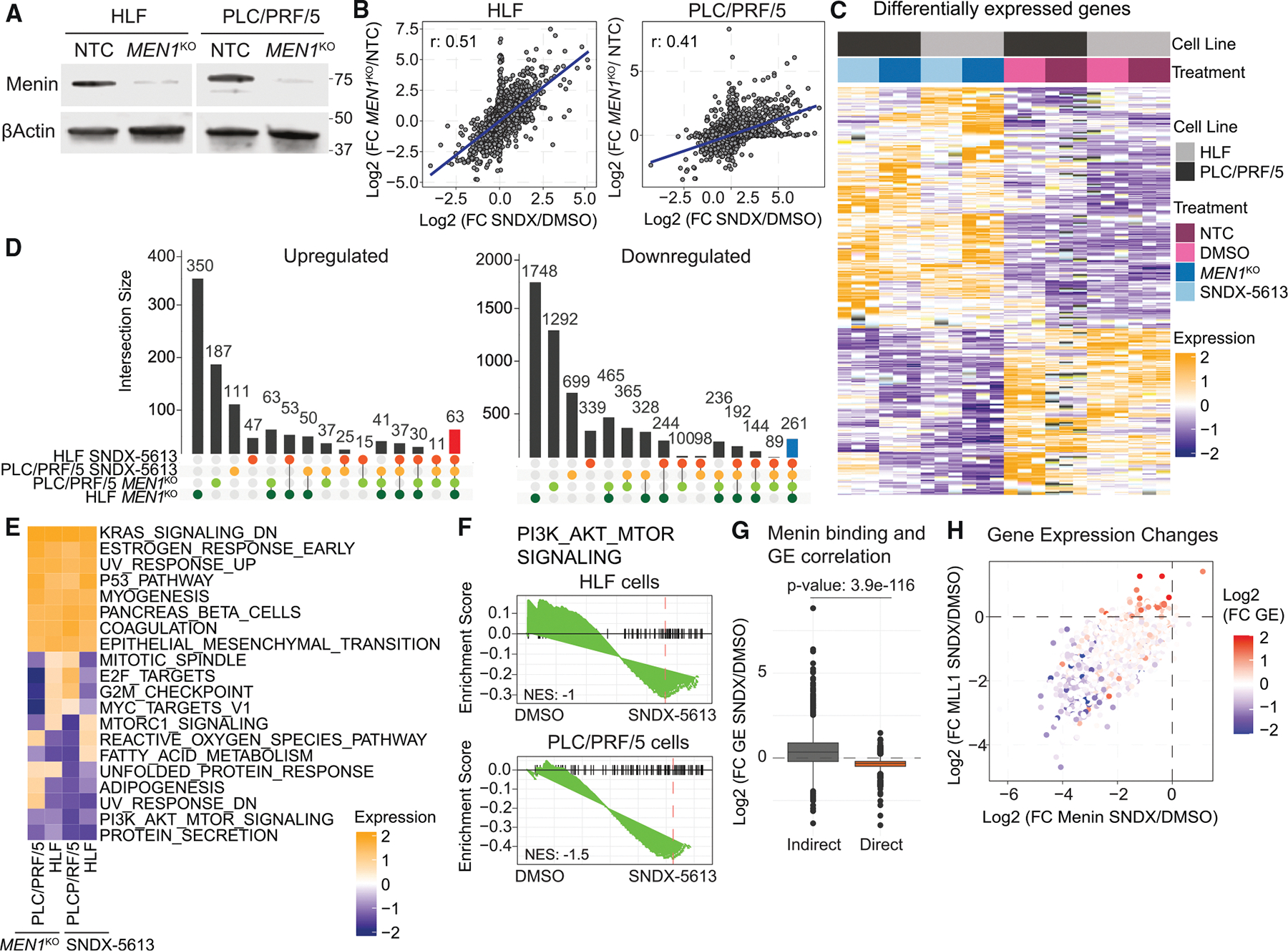
MEN1 gene knockout and the menin-MLL1 interaction inhibition affect similar signaling pathways in HCC (A) Immunoblotting against menin and β-Actin proteins in HLF and PLC/PRF/5 knockout (KO) and NTC cells. (B) Logarithmic fold changes between *MEN1* KO relative to NTC and SNDX-5613 treatment relative to DMSO from RNA-seq of HLF or PLC/PRF/5 cells. (C) Heatmap of differentially expressed genes in HLF and PLC/PRF/5 cells with *MEN1* or NTC KO or treated with SNDX-5613 or DMSO. (D) UpSet diagrams showing commonly up- and downregulated genes in HLF and PLC/PRF/5 cells treated with SNDX-5613 or upon *MEN1* KO. (E) GSEA ontologies with hallmark gene set as a reference affected by SNDX-5613 treatment and *MEN1* KO in HLF and PLC/PRF/5 cells. (F) GSEA analysis of gene expression changes in HLF or PLC/PRF/5 cells treated with SNDX-5613 with PI3K/AKT/mTOR signaling pathway from the hallmark gene set. (G) Logarithmic fold changes in gene expression and in menin and MLL1 binding to these genes in HLF cells treated with SNDX-5613. (H) Logarithmic fold changes in gene expression depending on menin-MLL1 binding in HLF cells treated with SNDX-5613.

To understand how disruption of menin alters gene expression, we performed RNA-seq in HLF and PLC/PRF/5 cells treated with 5 μM SNDX-5613 for 4 days, as in CUT&RUN experiments. In addition, we generated *MEN1*-KO cell lines using CRISPR-Cas9 (Figure 3A; Table S5). We observed a strong correlation between the fold changes of DMSO/SNDX-5613 and NTC/KO, with more genes downregulated in both cell lines (Figures 3B, 3C, S3A, and S3B). Differences between the drug and KO likely reflect the more severe effect of genetic ablation. This is consistent with our result showing that menin inhibition leads to widespread decreases in H3K4me3 occupancy at menin-MLL1-occupied promoters, prompting loss of gene activation. Comparing all conditions, we found 63 upregulated and 261 downregulated genes shared among SNDX-5613-treated and *MEN1*-KO cells (Figure 3D). We also observed a larger number of genes impacted by gene KO relative to menin inhibition. We noted that CRISPR-Cas9 KOs of menin were generally unstable, often resulting in a quick rebound of expression likely due to a small population of cells not genetically ablated that survived, while *MEN1* KO led to lethality.

We then investigated whether menin loss and inhibition affected pathways associated with cancer using the MSigDB database HALLMARK (H) and Oncogenic Signatures sets (C6).^32^ This revealed a consistent upregulation across both cell lines of epithelial-mesenchymal transition (EMT), KRAS-associated pathways, and estrogen response genes (Figures 3E, S3C, and S3D). We confirmed that upregulation of the EMT pathway by SNDX-5613 treatment enhanced cell migration of both HLF and PLC/PRF/5 cells (Figure S3E). Downregulated pathways were more variable, but consistent patterns emerged associated with cell cycle, PI3K, AKT, MYC, and YAP (Figures 3E and 3F). Both MYC and YAP have previously been shown to associate with menin in HCC.^33,34^

To investigate the direct impact of menin-MLL1 complex binding on the expression of potential target genes, we integrated CUT&RUN data with the RNA-seq results from HLF cells. We assigned genes as direct menin targets if a menin peak was located within the promoter and if menin binding decreased following menin inhibition and was also marked by loss of MLL1 and H3K4me3. This revealed that direct menin targets were downregulated, while indirect targets tended to be upregulated (Figure 3G; *p* = 3.9e−116), suggesting the upregulation of pathways we observed in gene set enrichment analysis (GSEA) was likely due to indirect effects of menin inhibition/loss (Figure 3E). Additionally, expression changes following menin inhibition correlated only moderately with menin binding, in contrast to MLL1 occupancy at menin sites (Figures S3F–S3H).

Directly comparing the correlation between menin and MLL1 binding and expression changes showed that this stringent assignment likely underestimates the number of genes impacted by the disruption of complex binding (Figure 3H). Together, these data suggest that menin-MLL1 complex disruption leads to numerous effects on gene expression and dysregulates key pathways involved in HCC cell survival.

### Menin inhibition causes loss of accessibility at menin-bound regions and alters chromatin accessibility at NF-YB motifs

**Figure 4.**
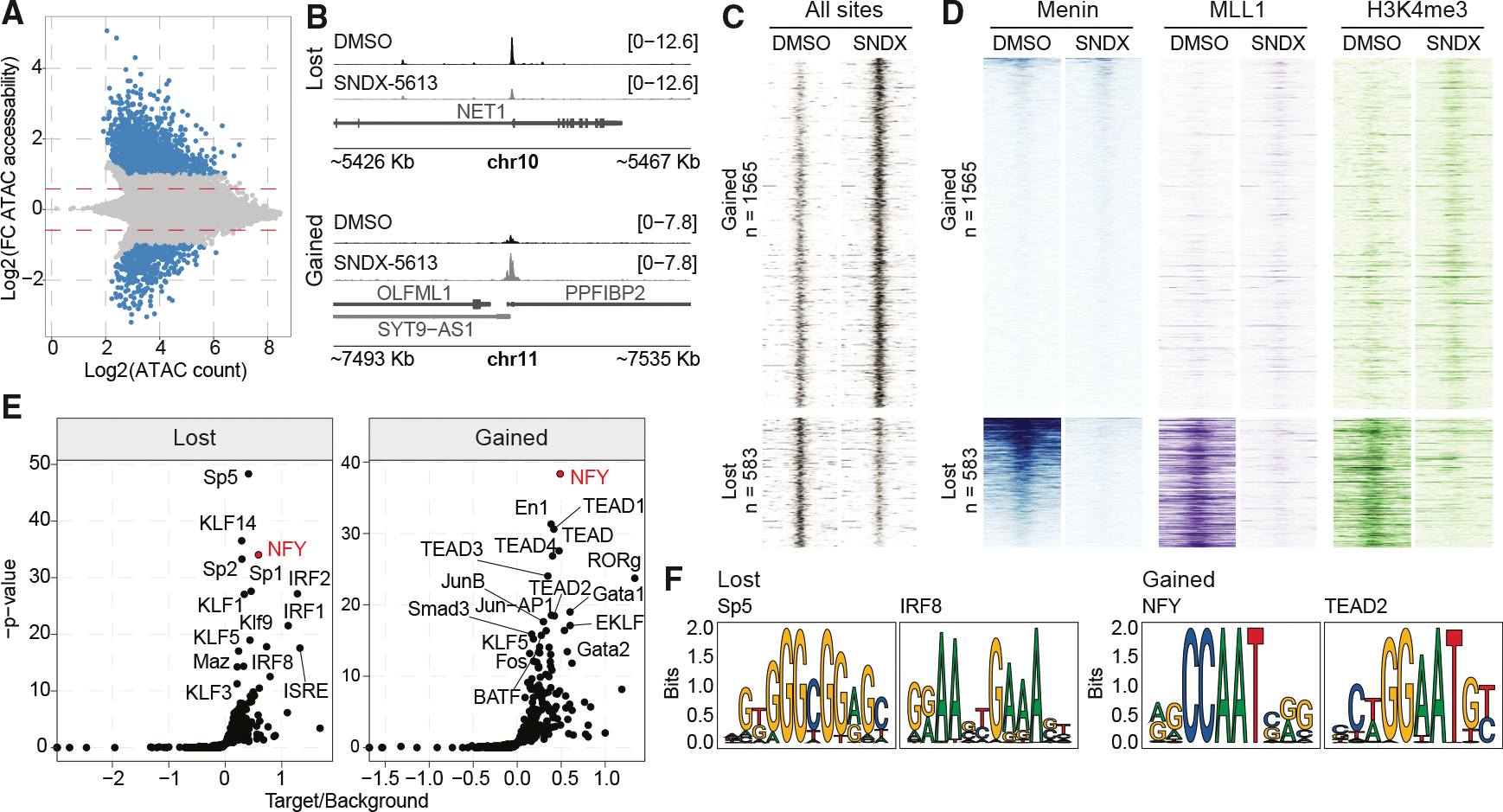
Menin inhibition affects chromatin accessibility in HCC cells (A) Logarithmic differential chromatin accessibility across ATAC-seq data from HLF cells treated with 5 μM SNDX-5613 for 4 days. (B) Representative sequencing tracks of ATAC-seq peaks at the promoter regions with lost and gained chromatin accessibility from HLF cells treated with SNDX-5613 for.4 days. (C and D) Heatmap showing open chromatin (C), or H3K4me3, menin, and MLL1 (D) ±2 kb from peak center at gained or lost ATAC-seq peaks from HLF cells treated with SNDX-5613. (E) Differential binding of transcriptional factors based on motif analysis of gained or lost ATAC-seq peaks relative to unchanged peaks in HLF cells upon SNDX-5613 treatment. (F) Examples of motifs derived from the ATAC-seq analysis with lost or gained chromatin accessibility.

To understand whether the menin inhibition impacts chromatin accessibility, we performed ATAC-seq in HLF cells treated with 5 μM SNDX-5613 for 4 days, as in the CUT&RUN and RNA-seq experiments. We identified 4,236 regions that gained and 2,534 regions that lost accessibility (*p*adj < 0.05, DESeq2) (Figures 4A and 4B; Table S6). Differentially accessible regions (DARs) with gained accessibility were associated with intergenic regions, introns, and promoters, while regions with lost accessibility were more associated with promoter and intronic peaks (Figure S4A). We further restricted our analysis to the 1,565 sites with log2 fold changes greater than 1.5 and 583 sites with log2 fold changes less than 1.5 to determine if changes in accessibility were associated with changes to menin, MLL1, or H3K4me3 occupancy at these DARs (Figures 4C, 4D, and S4B). This demonstrated a striking decrease in menin, MLL1, and H3K4me3 binding among the DARs with lost accessibility following menin inhibition. Very few gained sites had evidence of menin or MLL1 binding in either the DMSO- or SNDX-5613-treated conditions (Figure 4D). The data support a model where menin promotes open chromatin at transcriptionally active regions and that newly opened chromatin sites are independent of menin or MLL1 binding following menin inhibition.

To understand which TFs may be important for the altered chromatin accessibility at the newly opened sites, we used HOMER to search for motifs at gained or lost DARs relative to the stable accessible regions (*n* = 85,708). Lost sites were associated with NF-Y, KLF, SP, and IRF families (Figures 4E and 4F), and gained sites were associated with NF-Y, TEAD, and GATA motifs. We observed a similar increase in usage of TEAD and NF-Y motifs using TOBIAS to search for accessibility changes (Figure S4C). Given the enrichment of NF-Y family motifs in both gained and lost DARs, we hypothesized that NF-Y family members may redistribute in the genome and define a new transcriptional program. NF-Y contains three subunits (NF-YA, NF-YB, and NF-YC), which are all required for its function and have previously been shown to remain bound at menin target sites following menin inhibition and loss from chromatin in *MLL*-r AML.^35–37^

### NF-YB is a vulnerability in HCC cells following menin inhibition

**Figure 5.**
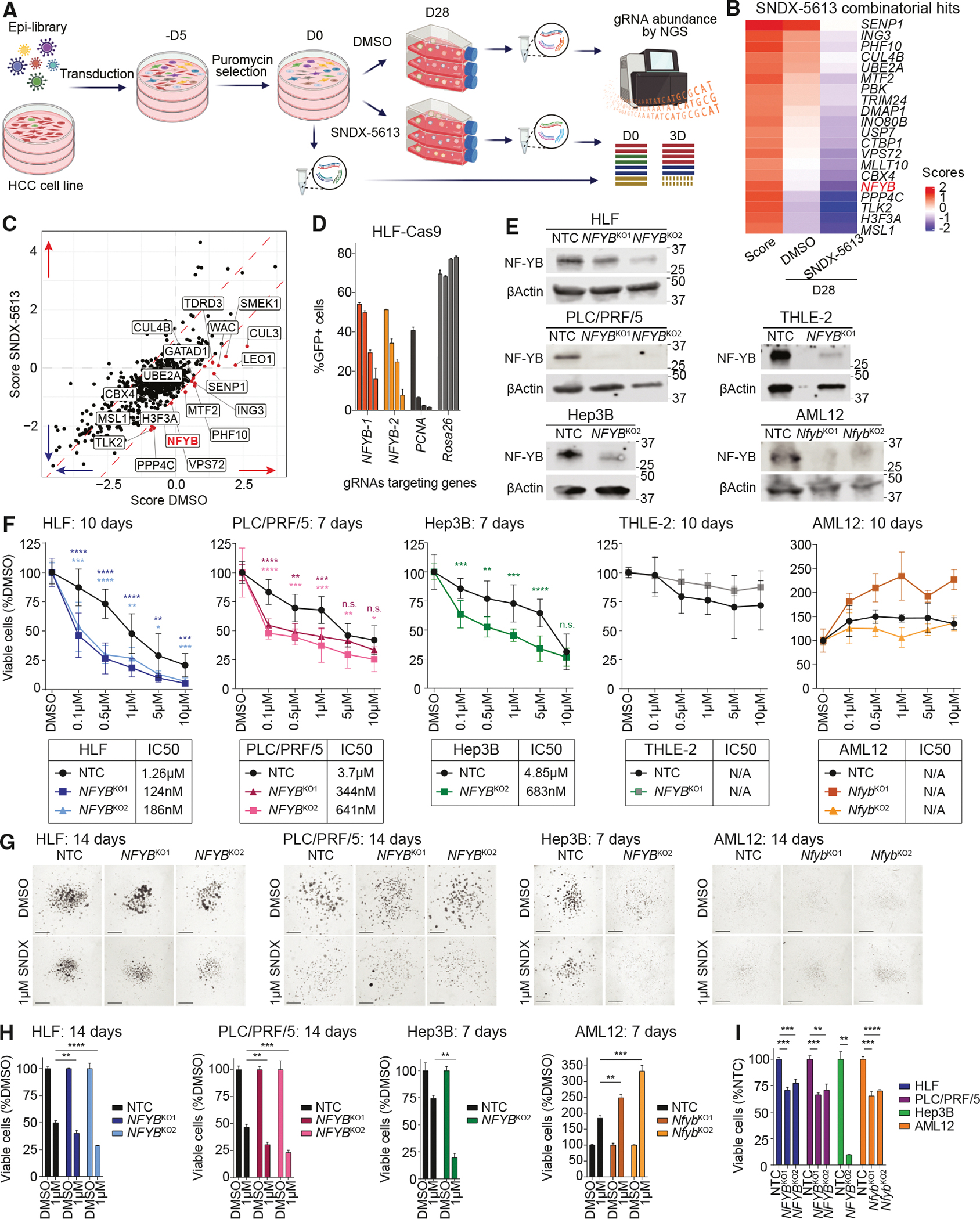
Menin inhibition combined with *NFYB* KO enhances HCC cell death (A) Scheme of epigenome-focused CRISPR-Cas9 screening in 3D settings in the presence of SNDX-5613 or DMSO. (B) Heatmap of potential menin inhibitor combinatorial targets from the CRISPR-Cas9 screen performed in HLF cells treated with 1 μM SNDX-5613 or DMSO for 28 days. The score was calculated as a subtraction of SNDX-5613 from DMSO at days 28/0. *NFYB* is shown in red. (C) Comparison of gene scores based on gene enrichment in DMSO- and SNDX-5613- (1 μM) treated groups from 3D CRISPR screens on HLF cells. (D) Summary of negative-selection CRISPR-Cas9 screen with sgRNAs targeting *NFYB* gene or with *PCNA* or *Rosa26* serving as the positive or negative controls, respectively, in 2D depicted at days 3, 14, 21, and 28. Bar graphs represent the mean of measurements of 3 independently transduced sets of cells using the same lentivirus. Error bars represent the SD. (E) Immunoblotting against NF-YB and β-Actin proteins in HLF-KO and NTC cells. (F) Cell viability of HLF, PLC/PRF/5, Hep3B, THLE-2, or AML12 cells with either *NFYB* KO or NTC upon SNDX-5613 treatment for 7 or 10 days, as indicated in 2D, assessed by the CTG assay. Error bars represent the SD of 3 independent experiments for HLF and PLC/PRF/5, 2 for Hep3B and THLE-2, or 1 for AML12 cells, each performed in 4 technical replicates. *p ≤ 0.05, **p ≤ 0.01, ***p ≤ 0.001, and ****p ≤ 0.0001 were calculated using a Student’s *t* test. (G and H) HCC and normal liver cell viability and growth after 7 or 14 days of SNDX-5613 treatment as indicated in 3D media. Cell growth and colony formation ability were assessed by microscopy (G), and cell viability was measured using the CTG assay (H). Micrographs were taken at ×1.25 amplification, with the bar indicating 1.3 mm. (I) Cell viability of HCC and normal liver cells with *NFYB* or NTC KO measured using the CTG assay. Bar graphs represent the mean of measurements of 3 (HLF, PLC/PRF/5, and AML12) or 2 (Hep3B) independently transduced sets of cells using the same lentivirus. Error bars represent the SD.

Next, to identify targets that synthesize HCC cells to menin inhibition, we performed CRISPR-Cas9 epigenome-focused screening in HLF cells treated with SNDX-5613. Following selection to remove non-transduced cells, we treated cells with DMSO or 1 μM SNDX-5613 and grew them in 3D conditions for 28 days (Figure 5A; Table S2). Comparing the gRNA abundance of each condition to its initial distribution revealed several targets that increased sensitivity to menin inhibition. *NFYB* gene KO was among the strongest SNDX-5613-associated hits, consistent with our hypothesis that it relocalizes in the genome following menin inhibition to support the expression of pro-growth genes (Figures 5B and 5C). To validate these data, we first tested the essentiality of the *NFYB* gene in competitive growth assays. This revealed that NF-YB loss leads to decreased cell viability in HLF cells (Figure 5D). Next, we tested if combined *NFYB* gene KO and menin inhibition increases HCC cell death by generating polyclonal CRISPR-Cas9 lines targeting the *NFYB* gene with gRNAs (*NFYB*^KO^; Figure 5E). As the *NFYB* gene has previously been shown in DepMap24Q4 to be essential for most cancer cell lines, only a partial gene KO could be achieved in most of the tested cell lines. Treatment of *NFYB*^KO^ and NTC HCC cell lines (HLF, PLC/PRF/5, and Hep3B) with increasing concentrations of SNDX-5613 for 7 or 10 days in 2D revealed a significantly increased sensitivity to menin inhibition across the mid-range concentrations for all *NFYB*^KO^ cell lines (Figure 5F). Normal human THLE-2 and murine AML12 liver cell lines used as a negative control have not shown similar sensitivity to combined *NFYB*-KO and SNDX-5613 treatment. We observed similar increased sensitivity of menin inhibition to *NFYB* gene KO in HCC cell lines and increased cell growth in the control AML12 cell line in 3D conditions (Figures 5G and 5H). We have also confirmed the essentiality of the *NFYB* gene in all tested cell lines in 3D settings (Figure 5I). Although we have not observed significant changes in apoptosis or the cell cycle with the combined treatment, we did detect accumulated cell debris in our 3D culture conditions, which might indicate cell death through various non-programmed mechanisms (Figures 5G, 5H, S5A, and S5B). Altogether, these data support the importance of the menin-MLL1 and NF-Y complex cooperation for HCC survival.

### NF-YB relocalizes following menin inhibition

**Figure 6.**
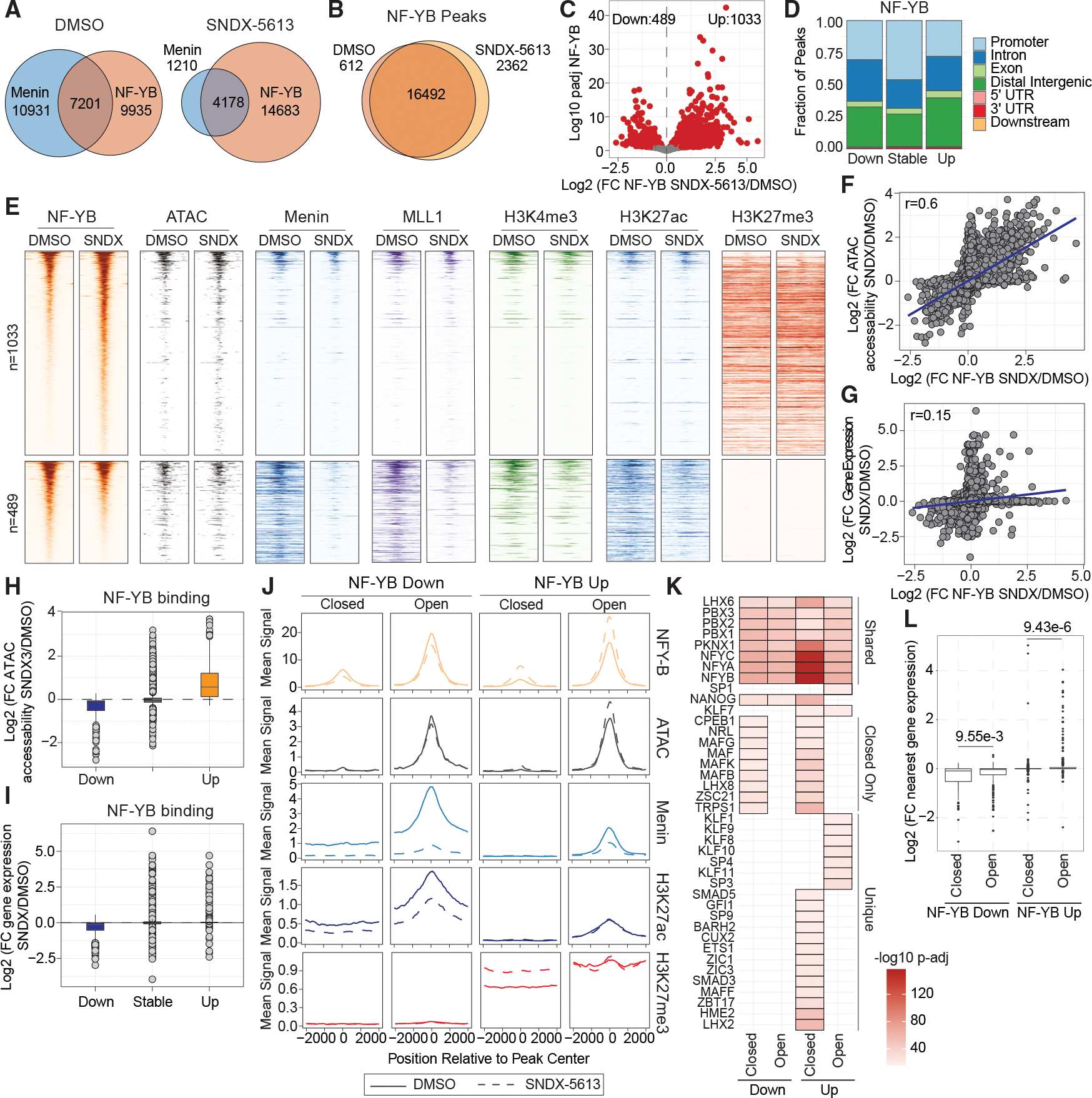
NF-YB binding correlates with newly opened chromatin sites in HCC cells (A and B) Venn diagram of the overlap between NF-YB and menin peaks (A) or NF-YB peaks alone (B) in HLF cells treated with DMSO or SNDX-5613. (C) Volcano plot of logarithmic differential binding of NF-YB in HLF cells upon SNDX-5613 treatment. (D) Distribution of binding sites relative to genomic features for NF-YB based on its binding status (lost, stable, or gained). (E) Heatmaps showing the CUT&RUN or ATAC-seq signal in a ±2-kb window for NF-YB, menin, MLL1, H3K4me3, H3K27ac, and H3K27me3 from HLF cells treated with SNDX-5613 at NF-YB gained or lost peaks. (F and G) Logarithmic fold changes between NF-YB binding and chromatin accessibility (F) or gene expression (G) upon SNDX-5613 treatment relative to DMSO in HLF cells from CUT&RUN and ATAC-seq data (F) or RNA-seq data (G). (H and I) Logarithmic fold change of chromatin accessibility (H) or gene expression (I) based on the NF-YB binding status (lost, stable, or gained). (J) Metaplots showing the normalized signal in a ±2-kb window of NF-YB sites for ATAC-seq chromatin accessibility, menin, H3K27ac, and H3K27me3 across CUT&RUN-seq data from HLF cells treated with SNDX-5613. (K) Motif enrichment of sites that either gain or lose NF-YB and are found in open or closed chromatin. (L) Expression of the nearest gene to NF-YB peaks from (K) in HLF cells treated with SNDX-5613.

To test whether NF-YB changes localization after menin inhibition, which may help HCC cells to survive, we performed CUT&RUN for NF-YB protein following 4 days of treatment with 5 μM SNDX-5613. In DMSO-treated conditions, ~40% of NF-YB peaks overlapped with menin peaks (7,201/9,935; *p* = 0; hypergeometric test). Following treatment with SNDX-5613, the remaining menin sites overlapped ~77% of NF-YB sites (4,178/5,388; *p* = 0; hypergeometric test). However, following treatment, most NF-YB peaks did not overlap with menin peaks (4,178/18,861, 22%; Figure 6A). By peak overlap, we detected 612 NF-YB peaks lost and 2,362 peaks gained upon SNDX-5613 treatment (Figure 6B). A more stringent analysis using DESeq2 to identify significantly distinct differentially occupied regions (DORs) revealed 489 lost sites (lost), 1,033 gained sites (gained), and 18,780 sites that were not statistically different (stable) (Figure 6C; *p*adj < 0.05). Stable sites were predominantly found at promoters, while altered sites were more likely to be found in an intron or distal region (Figure 6D). Notably, ~40% of NF-YB sites did not overlap at ATAC-seq peaks in either the DMSO- or SNDX-5613-treated conditions (Figure S6A). This was unlikely caused by non-specific binding or technical issues in CUT&RUN, as the most enriched motif in NF-YB peaks, independent of ATAC-seq peaks, was the NF-Y motif (Figure S6B). These data are also consistent with prior reports that NF-Y could bind closed chromatin or inaccessible regions through a unique histone-like mechanism.^37–39^

Next, we assessed if the loss or gain of NY-YB binding led to additional changes in chromatin accessibility (Figure 6E). We noted that chromatin accessibility was generally decreased at NF-YB lost sites and increased at newly bound NF-YB sites. Additionally, we found that NF-YB loss was also associated with decreased menin and MLL1 occupancy (Figures 6E and S6C). Furthermore, NF-YB gained sites did not show a gain in menin or MLL1 binding, suggesting that the changes in chromatin accessibility at these sites are not dependent on the menin-MLL1 complexes (Figure S6C). Most gained NF-YB sites did not overlap a menin binding site (53/1,565), while sites that lost NF-YB overlapped a menin binding site ~50% of the time (279/581; Figure S6D). We observed similar changes in H3K4me3 and H3K27ac occupancy at NF-YB sites, further supporting that the primary cause of altered gene expression at sites losing menin and MLL1 is a general decrease in chromatin accessibility associated with combined menin, MLL1, and NF-YB loss, leading to an inactive chromatin state (Figure S6C).

Our data also revealed that gained NF-YB sites, although associated with increased chromatin accessibility, were still generally embedded in domains of H3K27me3 (Figures 6E and S6C), consistent with prior studies showing NF-Y binding in both active and repressed chromatin regions.^40^ These data suggest that the sites with gained NF-YB binding may reflect an early activation state that has not fully de-repressed chromatin. Consistent with this, we found a strong correlation between the fold change of NF-YB occupancy and chromatin accessibility (Figures 6F–6H; r = 0.6, *p* = 0). However, the expression of the nearest gene to these sites was not as strongly correlated, and most gained NF-YB sites were not associated with a concomitant increase in gene expression (Figures 6G–6I; r = 0.15, *p* = 7.4e−69). We performed a similar analysis of differential occupancy specifically at accessible chromatin regions (ATAC-seq peaks) and observed that NF-YB binding changes were concordant with chromatin accessibility and, to a lesser extent, with gene expression changes (Figures 6H, 6I, S6E, and S6F). Together, these data support a model where NF-YB contributes to menin-dependent gene activation through co-localization. Upon menin inhibition, NF-YB relocalizes to novel repressed sites that are poised for future activation while remaining at some sites in the absence of menin.

We further analyzed the regions that gain NF-YB binding following menin inhibition. We separated sites by gain or loss of NF-YB binding and by their overlap with an ATAC-seq peak (open vs. closed chromatin). We hypothesized that different mechanisms or biological processes might be associated with these four categories. Sites that increase NF-YB binding were enriched for H3K27me3, even when those sites were also in open chromatin and increased in accessibility, which was also observed using ontology analysis of the nearest gene (Figures 6J and S6G). Using the MEME software suite, we identified TF motifs that were either common across all groups or unique to one or more categories (Figure 6K). As anticipated, NF-Y motifs were the most enriched in all four groups. Additionally, we found that members of the minor-allele frequency (MAF) family were enriched at chromatin-inaccessible sites. Upon SNDX-5613 treatment, we observed that decreased gene expression was more significant at NF-YB lost regions in closed chromatin compared to those in open chromatin. Conversely, increased gene expression at NF-YB sites was associated with its gained binding in open chromatin vs. closed chromatin (Figure 6L). Together, these data suggest that distinct TFs cooperate with NF-Y to mediate functions at these sites.

### NF-Y complex primes activation of alternative pathways upon menin inhibition

**Figure 7.**
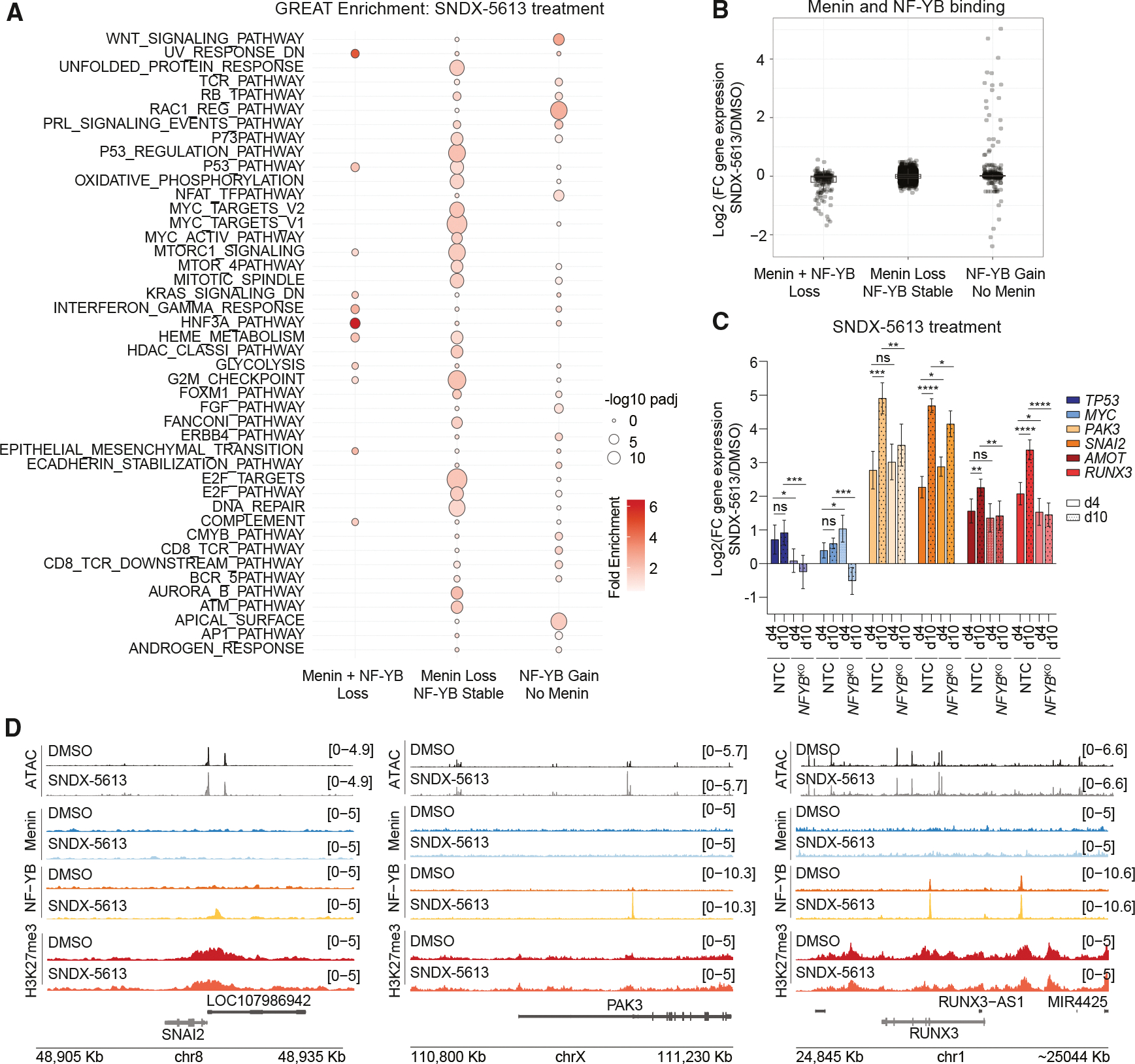
NF-YB relocalization activates new molecular pathways (A) GREAT enrichment analysis of menin and NF-YB direct targets based on their binding status (lost, stable, or gained) using the hallmark gene set comparing HLF cells treated with SNDX-5613 to those treated with DMSO. (B) Logarithmic differential gene expression analysis based on menin and NF-YB binding status to the corresponding nearest gene in HLF cells treated with SNDX-5613 to DMSO. (C) mRNA expression in HLF NTC or *NFYB*^KO^ cells after 4 or 10 days of SNDX-5613 treatment (5 μM), as assessed by RT-qPCR and normalized to DMSO control. Bar graphs represent the mean of measurements of 2 technical replicates. Error bars represent the SD. *p ≤ 0.05, **p ≤ 0.01, ***p ≤ 0.001, and ****p ≤ 0.0001 were calculated using a Student’s *t* test. (D) Examples of CUT&RUN tracks showing distribution of ATAC-seq open chromatin, NF-YB, menin, and H3K27me3 binding in HLF cells treated with SNDX-5613.

One possible mechanism by which menin inhibition, combined with NF-YB loss, enhances cell death is that NF-Y drives a pro-survival transcriptional program following menin inhibition that remains bound by NF-Y and supports continued activation of pathways essential for HCC cell growth. To test this idea, we contrasted regions that lost both menin and NF-YB, lost menin but NF-YB remained stably bound, or, finally, had NF-YB gained peaks that were not previously associated with a menin peak. We used GREAT^31^ to associate these peaks with genes and determine if distinct gene expression programs were regulated by the different categories (Figure 7A). This analysis revealed that regions that lost both NF-YB and menin were associated with cancer pathways, such as p53 and KRAS signaling, as well as liver-specific TFs, such as HNF3A signaling. Regions that lost menin but retained NF-YB had strong enrichment for a wide range of cell growth pathways, such as FOXM, MYC, p53, and cell cycle. NF-YB gained regions independent of menin binding were uniquely associated with RAC1 and WNT signaling pathways. The expression of genes that lost both NF-YB and menin was also downregulated after menin inhibition (Figure 7B). The group that lost only menin led to minimal differences in expression, consistent with our hypothesis that these sites remain active and potentially support cell survival. Finally, there was a modest upregulation of genes that gained NF-YB but no menin at this early time point, suggesting that these sites may represent NF-Y priming of associated genes. Next, we evaluated if the expression of genes bound by NF-YB upon menin inhibition would be affected after longer exposure to the SNDX-5613. We revealed that the *TP53* and *MYC* genes bound by NF-YB that also lost menin-MLL1 showed a reduction in gene expression after 10 days of treatment with 5 μM SNDX-5613 with *NFYB* KO. Notably, in response to prolonged menin inhibition, newly bound NF-YB genes involved in cell proliferation, migration, and invasion in liver cancer (*PAK3*, *SNAI2*, *AMOT*, and *RUNX3*) showed a significant increase in expression, while *NFYB* KO reduced their expression (Figures 7C and 7D).^41–45^ Together, these data demonstrate that at certain menin-bound regions, NF-YB cooperates to support gene activation and gains new significance by continuing to support the expression of specific pathways and by relocalizing to prime new transcriptional targets.

## DISCUSSION

In this study, we used epigenome-focused CRISPR libraries to interrogate multiple HCC cell lines in both 2D and 3D culture conditions. Recent work has uncovered that 3D culture conditions can reveal distinct vulnerabilities and more closely mimic essentialities observed *in vivo*.^25^ While these approaches remain underutilized, our data support the idea that screening approaches should be expanded to include various growth conditions. Notably, we found that the loss of genes involved in protein ubiquitination had contrasting effects on cell growth between the two conditions. While these genes are classified as “common essential” in the DepMap24Q4 database, their loss promotes or is neutral for cell growth in 3D cultures, several of which have been shown to promote liver cancer growth (*HDAC3*, *KMT2D*, *CUL3*, and *CUL5*; Figures 1 and S1).^46–49^

We showed a dependency of HCC on the core subunits of the menin-MLL complex (Figure 1). The interaction between menin and MLL1 was initially found to be essential in *MLL*-r and *NPM1*-mutant AML subtypes.^21,50,51^ Subsequent studies have highlighted its significance in the carcinogenesis of solid tumors (prostate, breast, Ewing’s sarcoma, endometrial, head and neck, and liver cancer).^52^ Elevated menin expression in HCC has also been shown to correlate with poor survival prognosis and promotion of HCC development.^34^ However, the mechanism by which menin-MLL1 promotes HCC remained unclear, with reports suggesting *YAP1* or *PEG10* as critical drivers.^34^ With the recent success of clinical trials targeting the menin-MLL1 interaction in AML, understanding the functioning of the menin-MLL complex in HCC could shed light on its potential as a combination therapy for HCC.

Our study delineates multiple mechanisms by which menin contributes to cancer cell survival. Mechanistically, these differ in some important ways from *MLL*-r and *NPM1*-mutant AMLs, where a menin inhibitor partially depletes MLL1 from the genome. In HCC, we observe a nearly complete genome-wide depletion of both menin and MLL1 proteins from menin-bound regions, with consequent decreases of H3K4me3 and decreased expression at these target genes upon menin inhibition. These data support cancer-type-dependent functions of menin-MLL1 in HCC, with MLL1 generally unable to associate with chromatin in the absence of menin (Figure 2). We also have not observed menin-MLL1-complex-dependent *MEIS1*- and *PBX3*-driven gene expression found in *MLL*-r and *NPM1*-mutant AMLs. Instead, PI3K/AKT/mTOR signaling emerged as a downregulated signaling pathway upon menin inhibition, revealing a different mechanism behind HCC cell proliferation (Figure 3).^21^ Additionally, we found more sites that gained accessibility following menin inhibition, suggesting that disrupting menin-MLL1 leads to a novel transcriptional response. These sites were largely located away from active promoters defined by H3K4me3 and were not bound by menin or MLL1 in any condition, supporting a hypothesis that these genomic regions represented a menin-MLL1-independent response and potential resistance mechanism. We identified the motif of the NF-Y TF complex members in regions with altered chromatin accessibility (Figure 4). NF-YA has been shown to localize to the menin-bound regions in *MLL*-r AML,^36^ where the sites bound by NF-YA following the loss of menin also showed a recruitment of the histone H3 lysine 27 demethylase UTX. However, in *MLL*-r AML, it has not been previously observed that the NF-Y complex can relocate following menin inhibition. Our data suggested two additional mechanisms for NF-Y involvement in HCC: first, where menin loss also leads to the loss of NF-Y and second, where NF-Y could bind to novel regions, potentially activating new transcriptional programs. Mapping of NF-Y-bound sites following menin inhibition showed NF-Y loss from some menin-occupied loci, and these correlated with decreased expression. The sites that gained NF-YB in the absence of menin correlated with moderate increases in accessibility and gene expression (Figure 6), potentially indicating an early response to menin inhibition. While our early time point allowed us to separate the cell death effect from the chromatin and transcriptional consequences, longer exposure to menin inhibitors may uncover NF-Y-dependent activation. NF-Y has a unique histone-like motif that allows it to bind closed chromatin. We uncovered distinct TF motifs in accessible compared to closed chromatin (Figure 6). The newly uncovered functions of the NF-Y complex upon menin inhibition might represent an HCC cell survival mechanism through either supporting oncogenic programs, such as MYC, p53, and E2F targets, or a novel activation of cell invasion and EMT associated with escape or metastasis (Figure 7). This mechanism of TF relocalization following therapy or during cancer progression may provide new opportunities for rational design of combinational therapies.

NF-YB was also among the strongest sensitizers to SNDX-5613 treatment in our 3D CRISPR-Cas9 screen, further supporting a role in driving survival mechanisms following the disruption of the menin-MLL1 interaction. Furthermore, the treatment of *NFYB*-KO cells with SNDX-5613 significantly decreased drug IC50 values, pointing to the necessity of the NF-Y complex for HCC cell survival upon menin inhibition (Figure 5). This combined effect may be due to NF-Y that remains bound to chromatin following menin-MLL1 disruption, which supports continued expression of specific pathways associated with cell growth or by disrupting later activation of novel escape mechanisms dependent on NF-Y. Thus, combinatorial treatment represents an appealing evolutionary trap to counteract resistance to menin inhibitors, especially in patients with *MEN1* mutations.

Together, our data demonstrate the first comprehensive multi-genomic analysis of menin-MLL1 complex function in HCC and its specific role in HCC cell growth. We demonstrate that NF-Y is a critical modulator of menin-MLL1 function and that both complexes cooperate to maintain an essential HCC survival expression program.

### Limitations of the study

Only a few publicly available HCC cell lines do not carry a *TP53* mutation. Considering that patients with liver cancer often have wild-type *TP53* status, this creates difficulty in evaluating whether menin inhibition would show a similar strong anti-tumor effect in HCC cells with wild-type *TP53* status.

As there is no publicly available inhibitor for the NF-Y complex or NF-YB subunits specifically, for our combinatorial studies, we had to evaluate the cooperation between the SNDX-5613 treatment and *NFYB* genetic KO. *NFYB* KO has been previously shown to be essential for most cancer cell lines (DepMap24Q4), so separating the effect of protein loss and its inability to bind to chromatin was not possible.

We also found evidence of TFs contributing to NF-Y complex binding. However, many TF families share similar motifs. Therefore, a more detailed analysis is needed to uncover which family members cooperate with NF-Y to drive specific molecular programs upon menin inhibition, which cannot be determined from a motif analysis alone.

## RESOURCE AVAILABILITY

### Lead contact

Requests for further information and resources should be directed to and will be fulfilled by the lead contact, Jesse R. Raab (jraab@med.unc.edu).

### Materials availability

Plasmids generated in this study are available from the lead contact with a completed materials transfer agreement.

### Data and code availability

- Data for CUT&RUN, RNA-seq, ATAC-seq, and CRISPR are available under GEO accession numbers GEO: GSE293692, GSE293693, GSE293691, and GSE293690. Processed data for each experiment are included as supplemental information. CRISPR screen scores and per gene counts can be found in Table S2.
- Code for pre-processing CRISPR, RNA-seq, CUT&RUN, and ATAC is available at http://github.com/raab-lab/, and code used for the analysis of data is available at http://github.com/raab-lab/dzama2025-menin-mll.
- Any additional information required to reanalyze the data reported in this paper is available from the lead contact upon request.

## STAR★METHODS

### EXPERIMENTAL MODEL AND STUDY PARTICIPANT DETAILS

#### Cell lines

All HCC (HLF (RRID:CVCL_2947), PLC/PRF/5 (RRID:CVCL_0485), SNU398 (RRID:CVCL_0077), and SNU449 (RRID:CVCL_0454)), murine normal liver AML12 (RRID:CVCL_0140) and Lenti-X 293 (RRID:CVCL_0045) cell lines were purchased from ATCC or were a gift as noted. HCC cell lines are male. At time of purchase cell lines were tested for authenticity by ATCC and tested for mycoplasm upon arrival when freezing initially.

### METHOD DETAILS

#### Cell culture and drug treatment

Cells were cultured in a humidified incubator at 37°C with 5% CO2 for under 2 months. The corresponding medias as defined by manufacturer (DMEM, RPMI-1640, or DMEM:F12) supplemented with 10% FBS and 1% Penicillin-Streptomycin (Gibco), and, additionally for AML12 cells, 1× ITS Universal Culture Supplement (Corning). Trypan-excluding cells were plated at a density of 5,000–20,000 cells depending on a cell line per well in 1 mL media in 24 well plates. For CUT&RUN, ATAC-seq, and RNA-seq, cells were treated with DMSO or 5μM SNDX-5613 for 4 days unless otherwise indicated in the figure legend. Cells were split and replated in fresh media every 3–4 days if they were treated for longer than 4 days. Menin-MLL1 inhibitior (SNDX-5613) was purchased from MedChemExpress (HY-136175, CID: 132212657). For IC50 determination, cells were treated with small molecule inhibitor SNDX-5613 at 10μM, 5μM, 1μM, 500nM, and 100nM concentrations plus vehicle control (DMSO). Cell viability was assessed by CellTiter-Glo (CTG) luminescent cell viability assay (Promega) using CTG reagent for staining in a ratio with cells 1:1 for 10 min at RT with gentle agitation. The signal was assessed using fluorescence assay on Cytation 5 machine (BioTek).

#### Immunoblotting

HCC cells treated with SNDX-5613 compound and vehicle (DMSO) were lysed in RIPA buffer (1e6 cells/30 μL lysis buffer; 150 mM NaCl, 1.0% IGEPAL CA-630, 0.5% sodium deoxycholate, 0.1% SDS, 50 mM Tris, pH 8.0). Identical amounts of protein of each sample were loaded on a 4–15% TGX precast gels (BioRad), electrophoretically separated and transferred to PVDF membranes (BioRad) using a Trans-Blot Turbo machine (BioRad) according to the manufacturer’s protocol. The membranes were blocked with 1:1 ratio mix of Intercept blocking (LI-COR) and TBS (200 mM Tris, 1500 mM NaCl) buffers for 1h at RT. Membranes were cut to allow independent incubation with primary antibodies according to the manufacturer’s recommendations in the antibody mix with ratio 1:1:0.01 of Intercept blocking buffer, TBS buffer, and 10% Tween 20 overnight at 4°C with gentle agitation. The following antibodies were used: anti-H3K4me3 (RRID:AB_3076423), anti-Menin (RRID:AB_3678623), anti-MLL1 (RRID:AB_2891811), anti-NF-YB (RRID:AB_2549386), anti-Histone H3 (RRID: AB_10544537), anti-βActin (RRID:AB_2715534). Membrane then was washed 3 times with TBST buffer (TBS with 0.1% Tween 20) for 10 min at RT with gentle agitation. The membrane then was incubated with secondary antibodies according to the manufacturer’s recommendations in the antibody mix with ratio 1:2:0.01:0.002 of Intercept blocking buffer, TBS, 10% Tween 20, and 10% SDS for 1h at RT with gentle agitation. The following secondary antibodies were used: goat anti-rabbit 800 (RRID:AB_621843) and Goat anti-mouse 680 (RRID:AB_10706161). The membrane was developed using Odyssey CLx machine (LI-COR). The immunoblotting signal analysis was performed in ImageStudio (LI-COR).

#### CRISPR screen

CRISPR libraries were designed using the GUIDES web app (http://guides.sanjanalab.org/) using a list of genes collated by hand (Table S1). Oligos were synthesized by Twist and cloned into pLenticrsipr-V2 (RRID:Addgene_52961) as described. The lentivirus of gRNA library was made by transfecting HEK293T and 2:2:1 ratio of psPAX2:pLenticrispr:pMD2.G (RRID:Addgene_12260, RRID:Addgene_12259) before harvesting viral supernatant at 72h. Cells for screen were infected at a multiplicity of infection of 0.25–0.3, selected for 7 days with 1μg/mL puromycin for non-infected cells. Cell pellets from ≥5e6 cells were harvested for genomic DNA (gDNA) using Monarch Genomic DNA Purification Kit. At least 1.5e6–3e6 cells was then placed under selective pressure (2D or 3D growth, or with 1μM SNDX-5613) for 28 days. This represents >500× coverage of the CRISPR library. After 28 days of growth, gDNA was harvested as before and high-throughput sequencing libraries were generated by PCR with oligos used for adding Illumina adapters and barcodes. Libraries were sequenced 1 × 75bp on Nextseq 500.

#### CRISPR screen analysis

CRISPR/Cas9 screen data were processed using the MaGeCK pipeline using the non-targeting gRNAs as controls. Robust Ranking Algorithm (RRA scores) from each contrast (2D vs. Time 0; 3D vs. Time 0) in both cell lines were compared using R. For comparison of individual gRNAs counts were normalized for sequencing depth.

#### Competitive growth assays

gRNAs targeting genes of interest and control genes (PCNA and Rosa26) were cloned into GFP-gRNA-coupled Lenti_sgRNA_EFS_GFP (LRG) plasmid (RRID:Addgene_65656). Lentivirus was made from these plasmids as described above and used to infect HCC cell lines of interest such that ~50% of initial cells were infected. Percent of GFP+ cells was measured for up to 28 days and compared to GFP+ cells with either a *Rosa26* (non-targeting control) or *PCNA* (essential gene control) gRNA insertions.

#### qRT-PCR

1e6 cells were harvested in biological replicates and pelleted for 1 min at 500g at RT. Total RNA was isolated using the Monarch Total RNA Miniprep Kit including on column DNAse I digestion. Quantative real-time polymerase chain reactions (qRT-PCR) were carried out using SsoAdvanced Universal SYBR Green Supermix (BioRad) and run on a CFX96 Touch Real-Time PCR Detection System (Bio-Rad; RRID:SCR_018064). Relative gene expression was determined by the Δ/ΔCt value method and normalized to the internal control, *GAPDH*. Primer sequences are available on request.

#### Propidium iodide staining and cell cycle analysis

Propidium Iodide (PI) staining was performed as previously described.Cells were seeded at 70–80% confluency 24 h prior to staining with PI. Cells were detached using Accutase and counted to have ~1–10 million cells per sample in 5 mL of PBC. Cells were spun at ~300 xg for 6 min and resuspended in 500 μL PBS at a single cell suspension. 4.5 mL of cold 70% ethanol was added to each sample and incubated on ice at 4°C for 2 h. After the incubation, fixed cells were centrifuged at ~300 xg for 5 min. Cells were resuspended in 5 mL PBS, incubated for 1 min, then spun again at ~200 xg for 5 min. Cells were resuspended in 1 mL of PI staining solution (0.1% Triton X-100, 20 μg/mL PI, 200 μg/mL RNAse A) and incubated at 37°C for 15 min. Samples were processed on a Agilent NovoCyte Flow Cytometer using the default laser settings and a flow rate of 35 μL/min. Cells were gated based on their forward and side scatter and further gated based on their forward scatter height and area. Cell cycle analysis was done using the automated cell cycle analysis module from the NovoExpress software. This module fits gated cells to a model which calculates the approximate number of cells in each phase of the cell cycle based on DNA content.

#### Annexin V staining

Annexin V staining was performed as per manufacturer instructions (Thermo Fisher, A13201). In brief, cells were seeded at 70–80% confluency 24 h prior to staining. Cells were detached using Accutase to ensure minimal disruption to cell membranes. Cells were washed with cold PBS and resuspended in annexin-binding buffer (10 mM HEPES, 140 mM NaCl, 2.5 mM CaCl_2_, pH 7.4). Cells were counted and adjusted to a concentration of ~1 million cells/mL using annexin-binding buffer. 100 μL aliquots were removed from the adjusted suspension and added to a new microfuge tube. 5 μL of Annexin V Conjugate and 1 μL of 1:10 diluted LIVE/DEAD Fixable Far-Red Stain (Thermo Fisher, L34974) was added to each tube and incubated at room temperature for 15 min in the dark. Following the incubation, 400 μL of annexin-binding buffer was added to each sample and placed on ice. Samples were then processed on a NovoCyte Flow Cytometer with a flow rate of 66 μL/min and the following laser settings: APC = 375 and FITC = 300. Cells were first gated by their forward and side scatter and then further gated by their forward scatter height and area. Cell populations were identified based on their FITC area and APC area with the amount of Annexin V and Far-Red corresponding to four distinct cell populations.

#### CUT&RUN

CUT&RUN was adapted from Epicypher. 200,000–500,000 (depending on antibody) cells were pelleted for 3 min at 300g at room temperature (RT). Cells were washed twice in wash buffer (20mM HEPES pH 7.6, 150mM NaCl, 0.5mM Spermidine, protease inhibitor (1 tablet/30mL Roche). 10μL Concanavalin A beads (Bangs Labs, BP531) per sample were washed twice in Bead Activation Buffer (20mM HEPES ph7.9, 10mM KCl, 1mM CaCl_2_, and 1mM MnCl_2_). Cells were incubated with Concanavalin A beads in Wash buffer for 5–10 min at RT. Supernatant was removed and bead bound cells were resuspended in 50μL Wash buffer containing 0.05% Digitonin and 2mM EDTA. Antibodies were added to mixture and mixed by gentle flicking before incubation overnight at 4°C. The following antibodies were used: anti-H3K4me3 (RRID:AB_3076423), anti-H3K27me3 (RRID:AB_2616029), anti-H3K27ac (RRID:AB_2561016), anti-Menin (RRID:AB_3678623), anti-MLL1 (RRID:AB_2891811), anti-NF-YB (RRID:AB_2549386), and anti-IgG (RRID:AB_1550038). Bead bound cells were washed on a magnet 2 times using Wash buffer containing 0.05% Digitonin (Dig-Wash Buffer) before incubation in 50μL Dig-Wash buffer with guinea pig anti-rabbit secondary antibody (1:100, Novus NBP1–72763, RRID:AB_11024108) for 1 h at 4°C. Bead bound cells were washed as described above before incubation in 700ng/μL pAG-MNase (purified in-house per) for 1 h at 4°C. Bead bound cells were washed 4 times as above with cold Dig-Wash buffer then resuspended in 50μL cold Dig-wash buffer while kept on ice. 1μL of 100mM CaCl2 was added while on ice and cells were incubated on ice at 4°C for 30 min. Reaction was then quenched by addition of Stop buffer (340mM NaCl, 20mM EDTA, 4mM EGTA, 0.05μg/μL RNAseA (ThermoFisher, EN0531), and 0.1% Triton X-100). Cells were incubated at 37°C for 30 min in PCR machine before purifying DNA using a Zymo DNA Clean and Concentrator-25 (Zymo Research, D4014). Libraries were constructed using the Kapa Hyperprep Kit (Roche, 07962363001) with modifications. End-repair and A-Tailing reactions were incubated at 12°C for 15 min, 37°C for 15 min, and 58°C for 45 Minute. Adapters were ligated for 1 h using 5μL of 750nM adapter concentration (Roche Dual Index, 08861919702). Libraries were cleaned twice using 1.1× volumes Kapa pure beads before PCR amplification using Kapa Hifi PCR mix with the following conditions: 98°C for 30″ to denature then 14 cycles of 98°C for 15″, 60°C for 10″ before a final Kapa Pure Bead (Roche, 07983298001) clean-up using 1.2× bead volumes. Libraries were quantified and pooled before sequencing at 2 × 50bp or 2 × 150bp on an Illumina Nextseq 1000 or NovaSeq X plus10B.

#### CUT&RUN analysis

CUT&RUN data was first processed using our CUT&RUN Nextflow pipeline (github.com/raab-lab/cut-n-run Version 4.0) which implements alignment, filtering, sorting, peak calling, and creates bigwig files for visualization. Trimming was performed using trim_galore with default parameters. Alignment was performed using Bowtie2 –very-sensitive-local -X 800 parameters to hg38. Files were sorted and indexed using Samtools (RRID:SCR_002105) and duplicates were marked with Picard. Macs2 was used for peak calling (–call-summits qvalue = 0.05). Consensus peak calls across replicates were generated using the RMPSC package. Normalization for visualization was performed using an implementation of the “composition” method of CSAW to calculate normalization factors for each antibody before scaling individual tracks using this factor. Coverage tracks were generated using Deeptools (version 3.2.0). Final visualization was generated by averaging bigwig signal tracks across replicates. These steps are all implemented within the Nextflow pipeline raab-lab/cut-*n*-run. Downstream analysis was performed in R. Peaks were annotated to the nearest gene and genomic features using ChIPpeakAnno package. Heatmaps and metaplots of CUT&RUN signal were calculated using Deeptools computeMatrix and plotHeatmap functions. Motif analysis was performed using HOMER and TOBIAS.

#### RNA-seq

1e6 cells were harvested in biological replicates and pelleted for 1 min at 500g at RT. Total RNA was isolated using the Monarch Total RNA Miniprep Kit including on column DNAse I digestion. 750 ng of total RNA was used for mRNA library preparation using KAPA mRNA HyperPrep Kit (Roche, 08098123702) and in accordance with manufacturer’s instructions. The desired mean library size was selected in the range of 200–300 bp with fragmentation step for 6 min at 94°C. For adapter ligation, 7μM adapter stock was used, and 9 cycles were chosen for library amplification. Libraries were quantified and pooled before sequencing at 1 × 75bp on an Illumina Nextseq 1500.

#### RNA-seq analysis

FASTQ files were processed using our Nextflow pipeline (github.com/raab-lab/rnaseq) to generate count matrices for each transcript using Salmon. Differential expression analysis was performed by first importing and merging transcripts to genes using tximeta before DESeq2 (RRID:SCR_000154) to perform differential expression. Log2FoldChanges were shrunk using the lfcShrink(type = ‘apeglm’) function. All downstream plotting was performed using R. GSEA analysis was performed using clusterProfiler (version = 4.10.1, RRID:SCR_016884) with msidgdb^32^ to provide gene annotations.

#### ATAC-seq

ATAC-seq was performed using the Omni-ATAC protocol. Briefly, 50,000 cells were pelleted at 500 g × 5 minutes at 4°C. Cells were resuspended in 50uL of cold ATAC-RSB (10 mM Tris-HCl pH 7.4, 10 mM NaCl, and 3 mM MgCl_2_ in water) containing 0.1% IPEGAL-CA630, 0.1% Tween 20, and 0.01% Digitonin and pipetted up and down 3 times before incubating on ice for 3 min. The Lysis buffer was then washed out using 1mL cold ATAC-RSB containing 0.1% Tween 20 but no IPEGAL-CA630 or Digitonin. Nuclei were then pelleted at 500 g × 10 min at 4°C. All supernatant was removed, and pellet was resuspended in 50uL of transposition mix - TD buffer, 0.05% Digitonin, 0.1% Tween, and 200nM TN5 transposase (Diagenode, C01070012). Cells were incubated at 37°C for 30 min on a Thermomixer at 1000RPM. Reactions were cleaned using a Zymo DNA Clean and Concentrator-5 (D4014, Zymo Research). Libraries were amplified to add indexed adapters from under the following conditions. 72°C × 5 min, 98°Cx30″ followed by 5 cycles of 98°Cx10″, 63°Cx30″, 72°Cx1’. The amount of library was quantified using qPCR to select the correct total number of cycles as described in, before completing cycle as before. Final library was purified using a 2-sided bead clean up (Roche Kapa Pure Beads, Roche, 07983298001), with ratios of 0.6× (keeping the supernatant) and 1.2× (keeping the bound fraction) to remove large fragments and unincorporated adapters respectively. Libraries were then pooled and sequenced on a Nextseq 1000 (Illumina) 2 × 50bp.

#### ATAC-seq analysis

Fastq files were processed using our Nextlfow pipeline (github.com/raab-lab/cut-n-run –atac). This pipeline is similar to our CUT&RUN approach, with some ATAC-seq specific changes denoted by the –atac flag. Briefly, reads are aligned with Bowtie2, duplicates are marked with PICARD (RRID:SCr_006525), normalization factors are calculated using CSAW ‘efficiency’ approach on a consensus set of peaks from all replicates/conditions defined using RMSPC, as for CUT&RUN. For differential accessibility, reads overlapping consensus peaks were counted using the CSAW function regionCounts and then used as input for DESeq2 (RRID:SCR_000154) to perform differential testing and lfcShrink(type = ‘apeglm’). Annotations to nearest feature and gene were performed using ChIPpeakAnno (version).

### QUANTIFICATION AND STATISTICAL ANALYSIS

#### Statistical analysis

All computational analysis of genomics data was performed using the R programming language (version 4.3.2) unless otherwise noted. Individual packages and software used in each analysis are described in the previous sections. Statistical details can be found in the figure legends and text associated with each figure. Drug sensitivity and cell growth assays were analyzed in Prism GraphPad (version = 9, RRID:SCR_002798).

#### Rigor

Sample sizes were determined by standards for genomics assays. Randomization was not employed as each replicate consisted of a single dish of cells split into either treated or untreated groups for analysis. To minimize bias, we employed standardized analysis pipelines because blinding is not feasible for these types of analyses.

## Supplementary Material

1

2

3

4

5

6

7

SUPPLEMENTAL INFORMATION

Supplemental information can be found online at https://doi.org/10.1016/j.celrep.2025.116619.

## Figures and Tables

**KEY RESOURCES TABLE:** 

REAGENT or RESOURCE	SOURCE	IDENTIFIER

Antibodies		

MLL1 (D2M7U)	Cell Signaling Technology	RRID:AB_2891811
ASH2L (D93F6)	Cell Signaling Technology	RRID:AB_1950350
Goat anti-rabbit 800	Licor	RRID:AB_621843
Goat anti-mouse 680	Licor	RRID:AB_10706161
Guinea Pig anti-rabbit	Novus	RRID:AB_11024108
H3K4me3	Epicpyher	RRID: AB_3678623
H3K27me3	Cell Signaling	RRID:AB_2616029
H3K27ac	Active Motif	RRID:AB_2561016
Menin	Epicypher	RRID: AB_3678623
MLL1	Fortis	RRID:AB_2891811
H3	Cell Signaling	RRID: AB_10544537
Beta-actin	Cell Signaling	RRID:AB_2715534
NF-YB	Thermofisher	RRID:AB_2549386
IgG (DA1E)	Cell Signaling	RRID:AB_1550038

Experimental models: Cell lines		

HLF	Gift of Derek Chiang	RRID:CVCL_2947
PLC/PRF/5	ATCC	RRID:CVCL_0485
SNU449	ATCC	RRID:CVCL_0454
AML12	ATCC	RRID:CVCL_0140
Lenti-X 293	Takara	RRID:CVCL_0045
HepG2	ATCC	RRID:CVCL_0027
Hep3B	UNC Tissue Culture Facility	RRID:CVCL_0326
SNU398	Gift of Derek Chiang	RRID:CVCL_0077
THLE-2	ATCC	RRID:CVCL_3803

Chemicals, peptides, and recombinant proteins		

RNAse A	Thermo Fisher	EN0531
Propidium Iodide	Thermo Fisher	P3566
SNDX-5613	MedChemExpress	CID:132212657
cOmplete EDTA-free protease inhibitor	Roche	11873580001
Concanavalin A beads	Bangs Labs	BP531
pAG-MNase	Gift from Steven Henikoff, purified in-house	RRID: Addgene_123461
TN5	Diagenode	C01070012
Kapa Hyperprep DNA library Prep	Roche	07962363001
Kapa Hyperprep mRNA library prep	Roche	08098123702

Critical commercial assays		

Annexin V Conjugates for Apoptosis Detection	Thermo Fisher	A13201
Click-iT^™^ Plus EdU Alexa Fluor^™^ 488 Flow Cytometry Assay Kit Alexa Fluor^™^ 488	Thermo Fisher	C10632

Deposited data		

CUT&RUN	This Study	GSE293692
RNA-seq	This Study	GSE293693
ATAC-seq	This Study	GSE293691
CRISPR/CAS9 Screen	This study	GSE293690

Recombinant DNA		

LRG	Addgene	RRID:Addgene_65656
pLenticrispr-V2	Addgene	RRID:Addgene_5296
psPax2	Addgene	RRID:Addgene_12260
pMD2.G	Addgene	RRID:Addgene_1225
CRISPR Library	This Study	
Prism	Graphad	RRID:SCR_002798
R project for statistical computing	R-project	RRID:SCR_001905
DEseq2	Bioconductor	RRID:SCR_015687
Nextflow	Nextflow	RRID:SCR_024135
Deeptools	Deeptools	RRID:SCR_016366
clusterProfiler	Bioconductor	RRID:SCR_016884
Tidyverse	R-project	RRID:SCR_019186

## Highlights

- CRISPR screens reveal hepatocellular carcinoma dependency on menin-MLL1 complex
- Disruption of menin-MLL1 affects PI3K/AKT/mTOR pathway and chromatin accessibility
- Menin inhibition leads to NF-Y relocalization on chromatin linked to cell migration
- Combined menin inhibition and *NFYB* knockout enhances HCC cell death
