# Supplementary material for: Menin-MLL1 complex cooperates with NF-Y to promote hepatocellular carcinoma survival
Source: Cell Rep. Author manuscript; Available in PMC 2026 Jan 23. (PMC12829923; doi:10.1016/j.celrep.2025.116619)
Supplement: 1 [file NIHMS2132738-supplement-1.pdf]

Cell Reports, Volume *44*

Supplemental information

Menin-MLL1 complex cooperates with NF-Y to promote hepatocellular carcinoma survival

Margarita Dzama-Karels, Mallory Sokolowski, Peyton Kuhlers, Jacqueline A. Brinkman, John P. Morris IV, and Jesse R. Raab

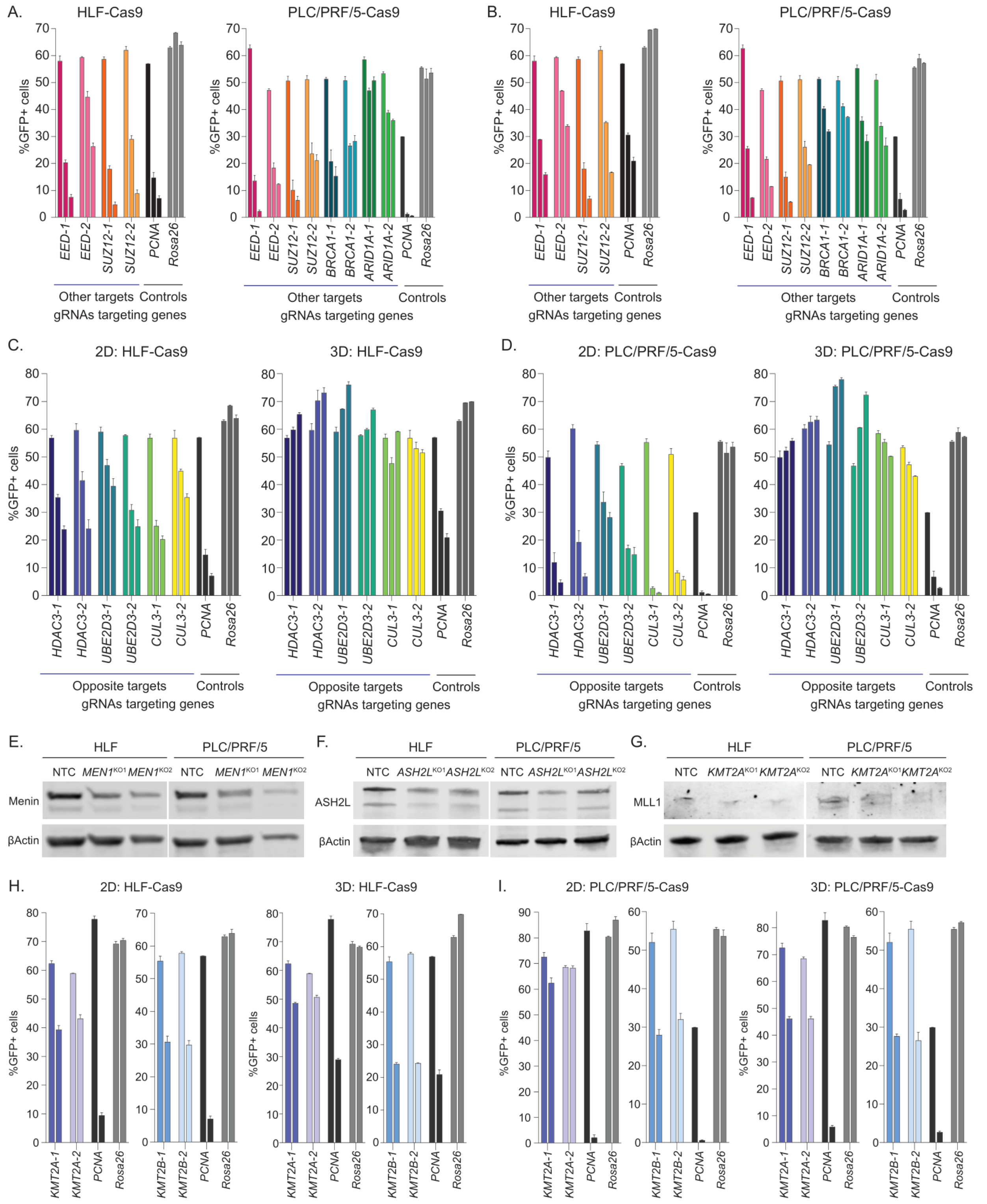

Figure S1. The negative selection CRISPR/Cas9 screen in 2D and 3D settings validates the initial CRISPR screen hits. (A-B) Summary of negative selection CRISPR/Cas9 screen with 2 sgRNAs targeting either *EED*, *SUZ12, BRCA1*, or *ARID1A* gene or 1 sgRNA targeting either *PCNA* or *Rosa26* serving as positive or negative controls, respectively, in 2D (A) and 3D (B) depicted at d3, d14, d21. Bar graphs represent the mean of measurements of 3 independently transduced set of cells using the same lentivirus.

Error bars represent SD. (C-D) Summary of negative selection CRISPR/Cas9 screen with 2 sgRNAs targeting either *HDAC3*, *UBE2D3,* or *CUL3* gene or 1 sgRNA targeting either *PCNA* or *Rosa26* serving as positive or negative controls, respectively, in HLF-Cas9 (C) or PLC/PRF/5 (D) cells in 2D and 3D depicted at d3, d14, d21. Bar graphs represent the mean of measurements of 3 independently transduced set of cells using the same lentivirus. Error bars represent standard deviation (SD). (E-G) Immunoblotting against menin (E), ASH2L (F), and MLL1 (G) with βActin as control in HLF and PLC/PRF/5 cells upon corresponding gene knockouts. (H-I) Summary of negative selection CRISPR/Cas9 screen with 2 sgRNAs targeting either *KMT2A* or *KMT2B* gene or 1 sgRNA targeting either *PCNA* or *Rosa26* serving as positive or negative controls, respectively, in HLF-Cas9 (H) or PLC/PRF/5 (I) cells in 2D and 3D depicted at d3 and d21. Bar graphs represent the mean of measurements of 3 independently transduced set of cells using the same lentivirus. Error bars represent SD.

A.

THLE-2	TP53	IC50 (uM)	Key Mutations	RRID	TP53 Impact
HLF	G244A	0.565	TERT	CVCL_2947	Pathogenic/Likely Pathogenic
PLC/PRF/5	R249S	<0.1	CDKN2AI;TSC2	CVCL_0485	Pathogenic/Likely Pathogenic
SNU398	S215I	0.123	CTNNB1	CVCL_0077	conflicting
SNU449	K239R;A161T	0.154	ARID1A; AXIN1	CVCL_0454	Pathogenic/Likely Pathogenic
Hep3B	NULL	0.56	AXIN;RB1	CVCL_0326	NA
HepG2	WT	0.538	NRAS;TERT	CVCL_0027	NA
THLE-2	WT	8	NA	CVCL_3803	NA
AML12	WT	NA	NA	CVCL_0140	NA

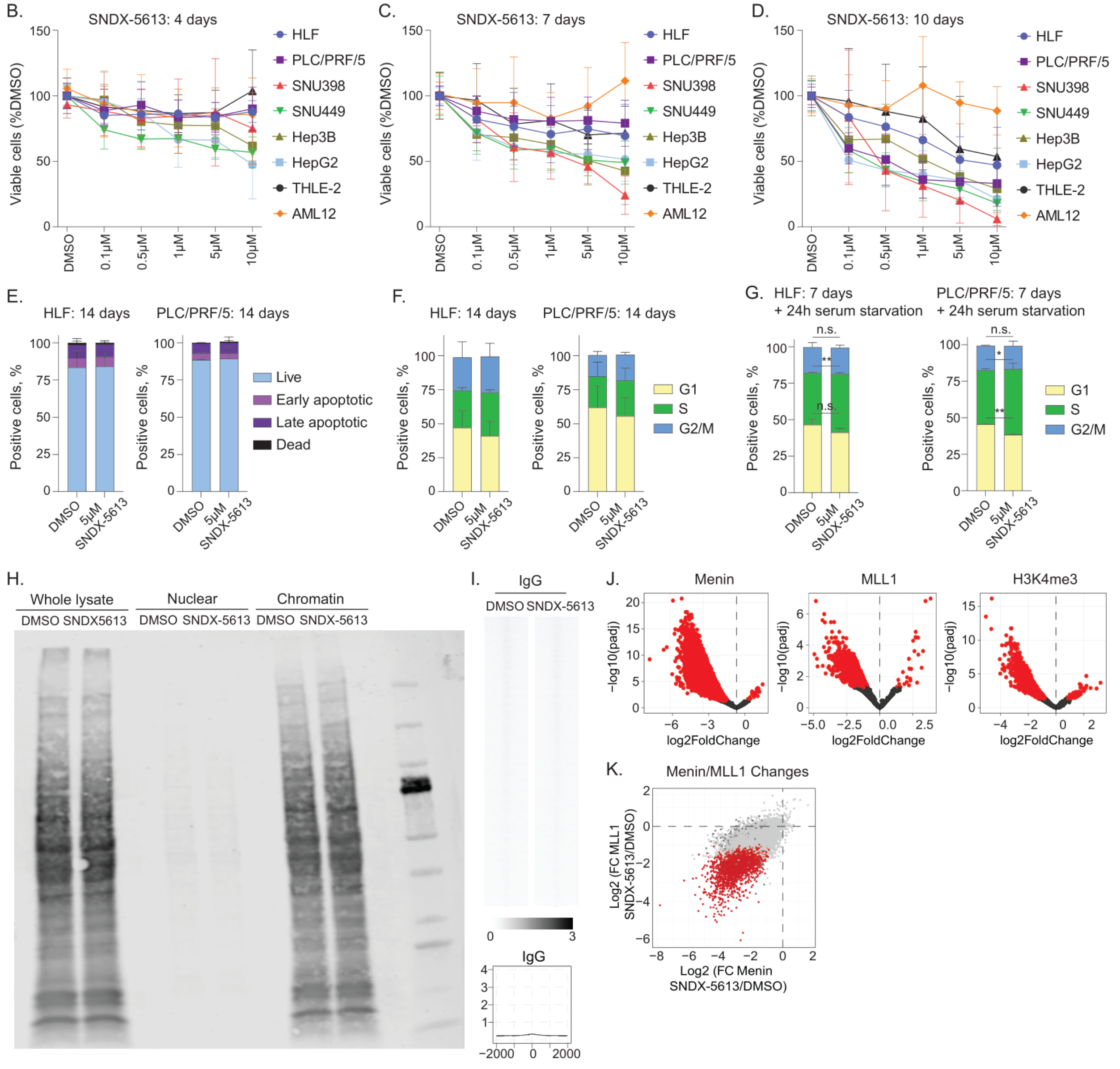

Figure S2. Inhibition of the menin-MLL1 interaction affects HCC cell survival and chromatin binding of the menin-MLL1 complex. (A) Summary of the *TP53* status and other key mutations in HCC and normal liver cell lines used for SNDX-5613 treatment. (B-D) HCC and normal liver cell viability following SNDX-5613 treatment assessed by Cell Titer Glo (CTG) assay after 4 (A), 7 (B) or 10 (C) days in 2D. Error bars

represent SD of 3 independent experiments, each performed in 4 technical replicates. (E) Percentage of apoptotic (annexin V) and dead (propidium iodide-stained) cells after treatment with 5μM SNDX-5613 treatment for 14 days. Error bars represent SD of 2 (HLF) or 1 (PLC/PRF/5) independent experiments, each performed in 3 technical replicates. (F-G) Propidium iodide and Click-IT EDU cell cycle analyses in HLF and PLC/PRF/5 cells treated with 5μM SNDX-5613 treatment for 14 days (F) or for 7 days with following serum starvation for 24h to synchronize the cell cycle (G). Error bars represent SD of 3 technical replicates in 2 (F) or 1 (G) independent experiments. P-values were calculated using Student t-test in GraphPad Prism version 9 software. *p-values ≤ 0.05, **p ≤ 0.01, ***p ≤ 0.001, ****p ≤ 0.0001. (H) Total protein stain for whole lysate, nuclear and chromatin fractions collected from HLF cells treated with DMSO or 5μM SNDX-5613 for 4 days, corresponding to H3K4me3 staining in Figure 2F. (I) Heatmaps showing the correlation of promoter peaks in a ±2-kb window with occupancy of IgG across CUT&RUN-seq data from HLF cells treated with 5μM SNDX-5613 for 4 days. IgG peaks are shown for all detected menin peaks. Bottom metaplot shows the mean of overall peak signals detected at the regions comparing DMSO and SNDX-5613 treatment conditions in HLF cells. (J) Differential occupancy analysis for menin, MLL1, and H3K4me3 performed at consensus peaks for each factor. (K) Logarithmic differential binding of menin and MLL1 proteins in HLF cells upon SNDX-5613 treatment (5μM).

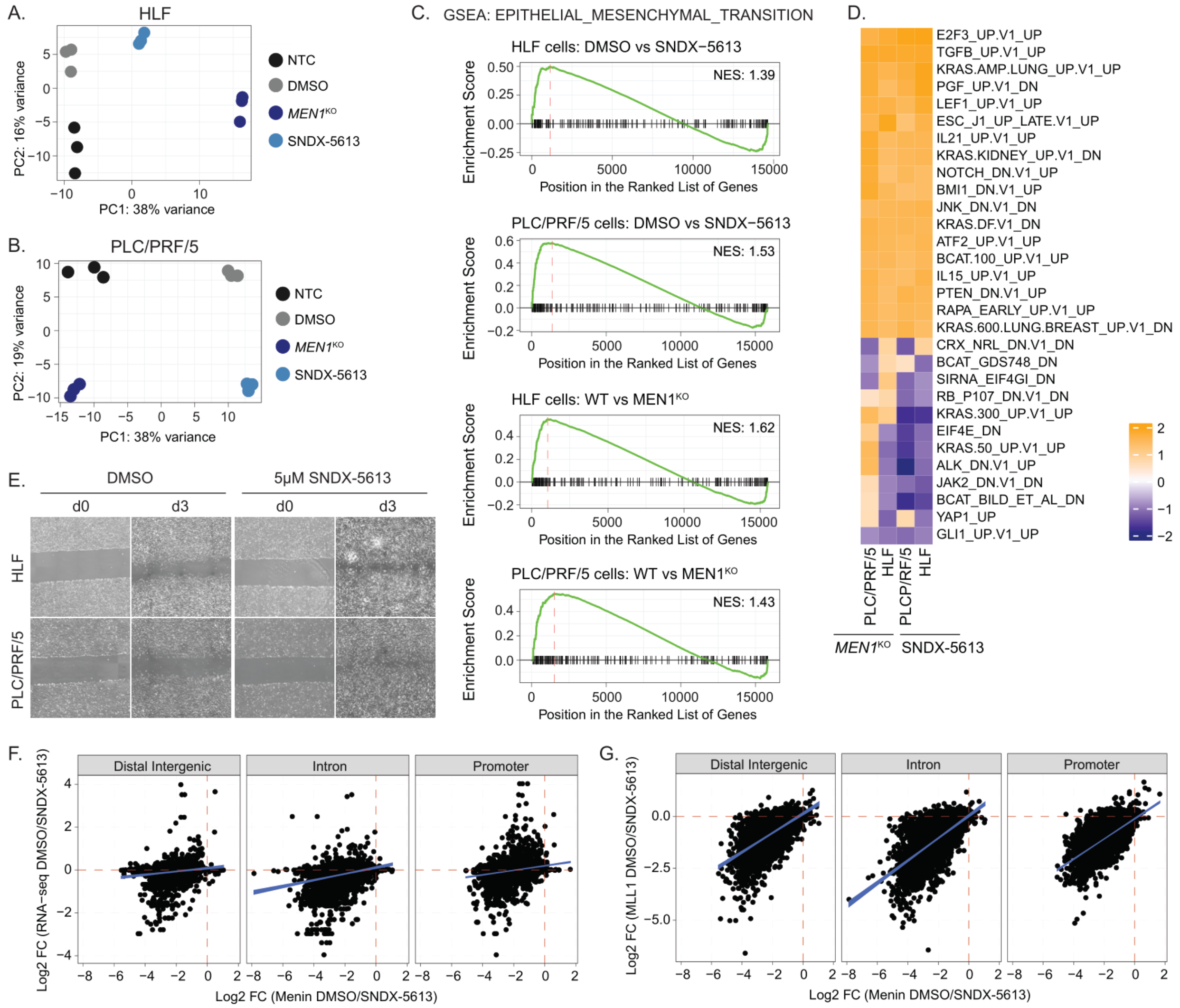

H.

	R²	Adj R²	Multiple Cor	RNA–MEN	MEN–MLL1
Distal Intergenic	0.026	0.025	0.162	0.159	0.527
Intron	0.089	0.089	0.299	0.281	0.556
Promoter	0.080	0.080	0.283	0.245	0.564

Figure S3. Menin inhibition alters gene expression programs. (A-B) PCA plots for replicates of HLF (A) or PLC/PRF/5 (B) cells either treated with 5μM SNDX-5613 or DMSO for 4 days or carrying a MEN1 or non-targeting gene knockout. (C) GSEA plots of gene expression changes in HLF and PLC/PRF/5 cell lines both either treated with 5μM SNDX-5613 or DMSO for 4 days or carrying a MEN1 or non-targeting gene knockout with Epithelial-Mesenchymal Transition pathway from hallmark reference gene set. (D) Comparison of GSEA ontologies with cancer gene set (C6) used as a reference affected by 5μM SNDX-5613 treatment and MEN1 knockout in HLF and PLC/PRF/5 cells. (E) Cell migration of HLF and PLC/PRF/5 cells upon DMSO or 5μM SNDX-5613 treatment for 7 days total with a scratch performed at 4 days of treatment. (F) Correlation of changes in gene expression and menin binding following 5μM SNDX-5613 for 4 days at menin peaks associated with different genomic features. (G) Correlation of changes in MLL1 binding with menin binding following 5μM SNDX-5613 for 4 days at menin peaks associated with different

genomic features. (H) Table of correlation metrics for menin-RNA expression and Menin-MLL for different genomic features.

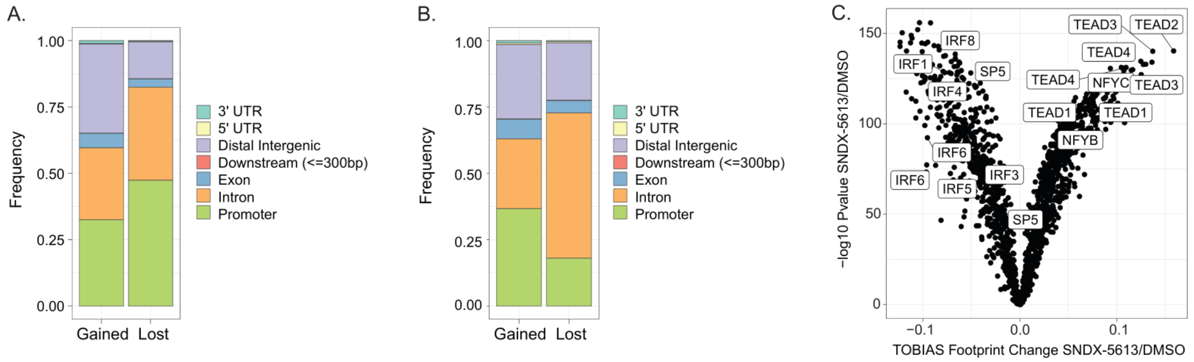

Figure S4. Chromatin accessibility at promoters and distal regions are disrupted by menin inhibition. (A-B) Genomic annotations associated with ATAC-seq peaks with increased or decreased accessibility of all sites (A) or filtered by fold change difference > 1.5 (B). (C) TOBIAS analysis of motif accessibility differences in HLF cells following 5μM SNDX-5613 treatment.

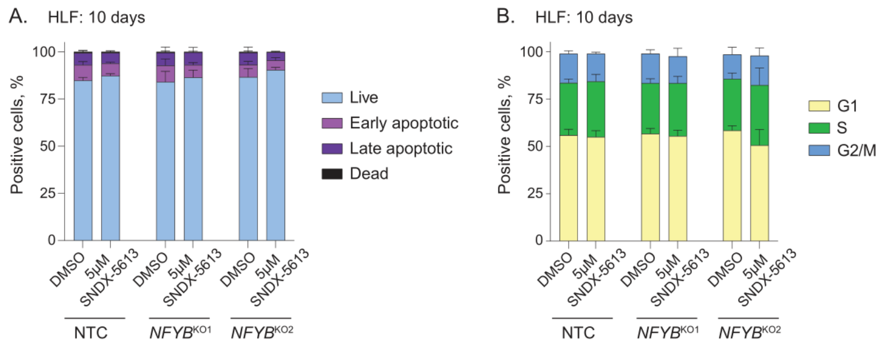

Figure S5. Combined menin inhibition and NFYB knockout does not significantly affect changes in apoptosis or cell cycle in HLF cells. (A) Percentage of apoptotic (annexin V) and dead (propidium iodide-stained) HLF cells with NTC or *NFYB* KO treated with DMSO or 5μM SNDX-5613 treatment for 10 days. Error bars represent SD of 2 independent experiments, each performed in 3 technical replicates. (F-G) Propidium iodide and Click-IT EDU cell cycle analyses in HLF cells with NTC or *NFYB* KO treated with DMSO or 5μM SNDX-5613 treatment for 10 days. Error bars represent SD of 3 independent experiments, each performed in 3 technical replicates.

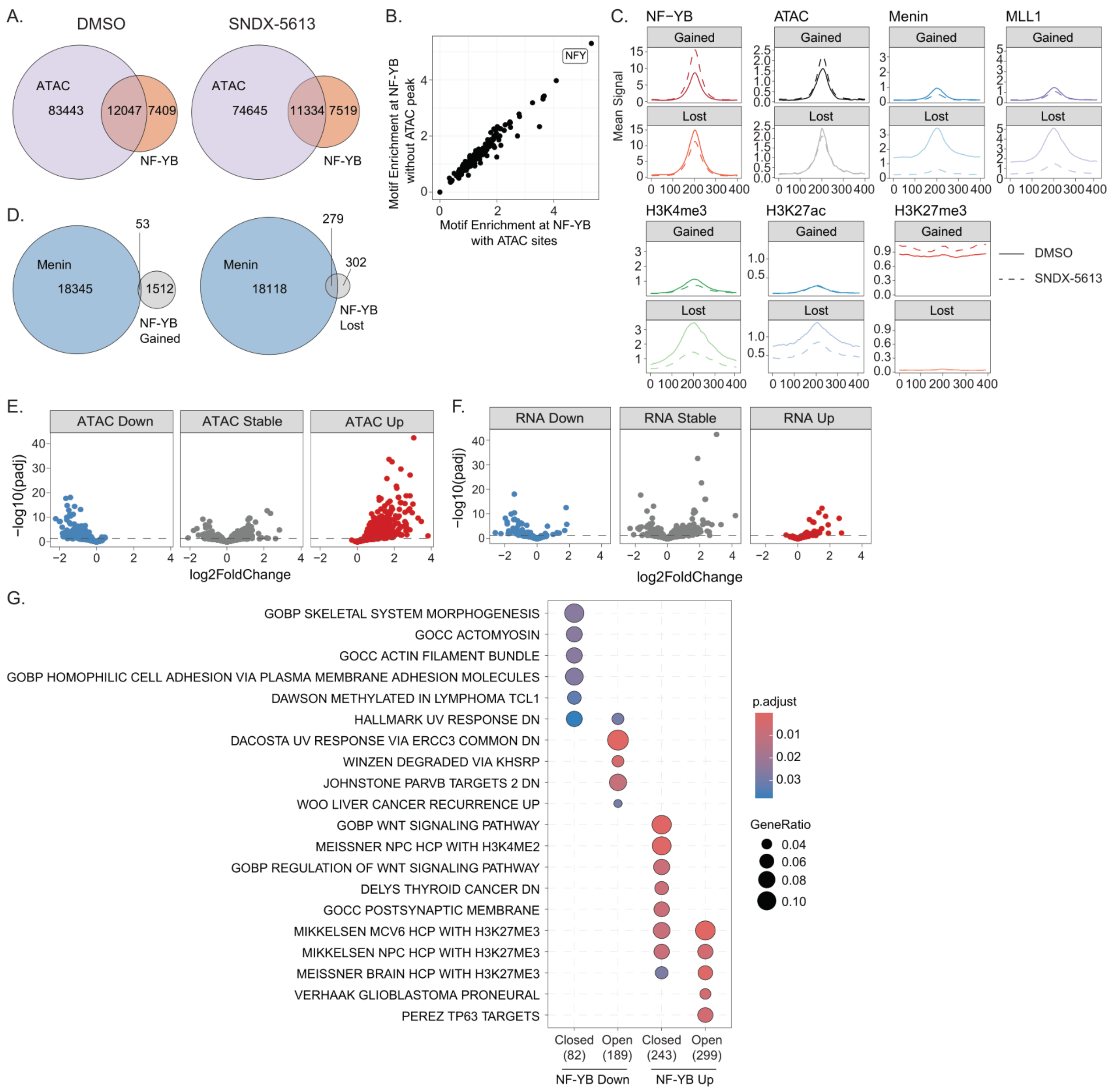

Figure S6. NF-YB is associated with altered chromatin accessibility at menin sites. (A) Venn diagram of overlap between NF-YB binding and ATAC-seq peaks in HLF cells treated with DMSO or 5μM SNDX-5613 for 4 days. (B) Motif analysis using HOMER at NF-YB peaks showing the overlap between NF-YB binding peaks that either overlap (x-axis) or do not with ATAC-seq peak (y-axis). (C) Metaplots showing the normalized signal of NF-YB, ATAC-seq chromatin accessibility, menin, MLL1, H3K4me3, H3K27ac, and H3K27me3 at NF-YB sites that gain or lose NF-YB in HLF cells treated with 5μM SNDX-5613 for 4 days. (D) Venn diagram of overlap between menin and NF-YB lost or gained binding in HLF cells treated with DMSO or 5μM SNDX-5613 for 4 days. (E) Differential logarithmic occupancy of NF-YB binding at ATAC-seq peaks faceted by whether the ATAC-seq peak was upregulated, downregulated or unchanged. (F) Differential logarithmic occupancy of NF-YB binding at sites associated with expression of the nearest gene to the peak. (G) Ontology of nearest gene to NF-YB bound regions based on the increased (UP) and decreased (DOWN) NF-YB binding and chromatin accessibility (open or closed) in HLF cells treated with 5μM SNDX-5613 to DMSO for 4 days using the MSigDB dataset with the Hallmark (gs_collection = H) and Canonical pathways and Chemical and Genetic Perturbations (gs_collection = C2) gene_set.
